# Extracting physiological information in experimental biology via Eulerian video magnification

**DOI:** 10.1186/s12915-019-0716-7

**Published:** 2019-12-12

**Authors:** Henrik Lauridsen, Selina Gonzales, Daniela Hedwig, Kathryn L. Perrin, Catherine J. A. Williams, Peter H. Wrege, Mads F. Bertelsen, Michael Pedersen, Jonathan T. Butcher

**Affiliations:** 1 Nancy E. and Peter C. Meinig School of Biomedical Engineering, Cornell University, 304 Weill Hall, Ithaca, NY 14853-7202 USA; 2 Department of Clinical Medicine, Aarhus University, Palle Juul-Jensens Boulevard 99, 8200 Aarhus N, Denmark; 3 California State University, 333 S Twin Oaks Valley Rd, San Marcos, CA 92096 USA; 4 Cornell Lab of Ornithology, Cornell University, 159 Sapsucker Woods Road, Ithaca, NY 14850 USA; 5 Center for Zoo and Wild Animal Health, Copenhagen Zoo, Roskildevej 32, 2000 Frederiksberg, Denmark; 6 Department of Veterinary Clinical Sciences, Faculty of Health and Medical Sciences, University of Copenhagen, Dyrlægevej 6, 1870 Frederiksberg C, Denmark; 7 Department of Bioscience, Aarhus University, C.F. Møllers Allé 3, 8000 Aarhus C, Denmark

**Keywords:** Videographic material, Signal processing, Heart rate, Respiration rate, Pulse wave velocity, Embryonic development, Infrasound

## Abstract

**Background:**

Videographic material of animals can contain inapparent signals, such as color changes or motion that hold information about physiological functions, such as heart and respiration rate, pulse wave velocity, and vocalization. Eulerian video magnification allows the enhancement of such signals to enable their detection. The purpose of this study is to demonstrate how signals relevant to experimental physiology can be extracted from non-contact videographic material of animals.

**Results:**

We applied Eulerian video magnification to detect physiological signals in a range of experimental models and in captive and free ranging wildlife. Neotenic Mexican axolotls were studied to demonstrate the extraction of heart rate signal of non-embryonic animals from dedicated videographic material. Heart rate could be acquired both in single and multiple animal setups of leucistic and normally colored animals under different physiological conditions (resting, exercised, or anesthetized) using a wide range of video qualities. Pulse wave velocity could also be measured in the low blood pressure system of the axolotl as well as in the high-pressure system of the human being. Heart rate extraction was also possible from videos of conscious, unconstrained zebrafish and from non-dedicated videographic material of sand lizard and giraffe. This technique also allowed for heart rate detection in embryonic chickens in ovo through the eggshell and in embryonic mice in utero and could be used as a gating signal to acquire two-phase volumetric micro-CT data of the beating embryonic chicken heart. Additionally, Eulerian video magnification was used to demonstrate how vocalization-induced vibrations can be detected in infrasound-producing Asian elephants.

**Conclusions:**

Eulerian video magnification provides a technique to extract inapparent temporal signals from videographic material of animals. This can be applied in experimental and comparative physiology where contact-based recordings (e.g., heart rate) cannot be acquired.

## Background

**Fig. 1:**
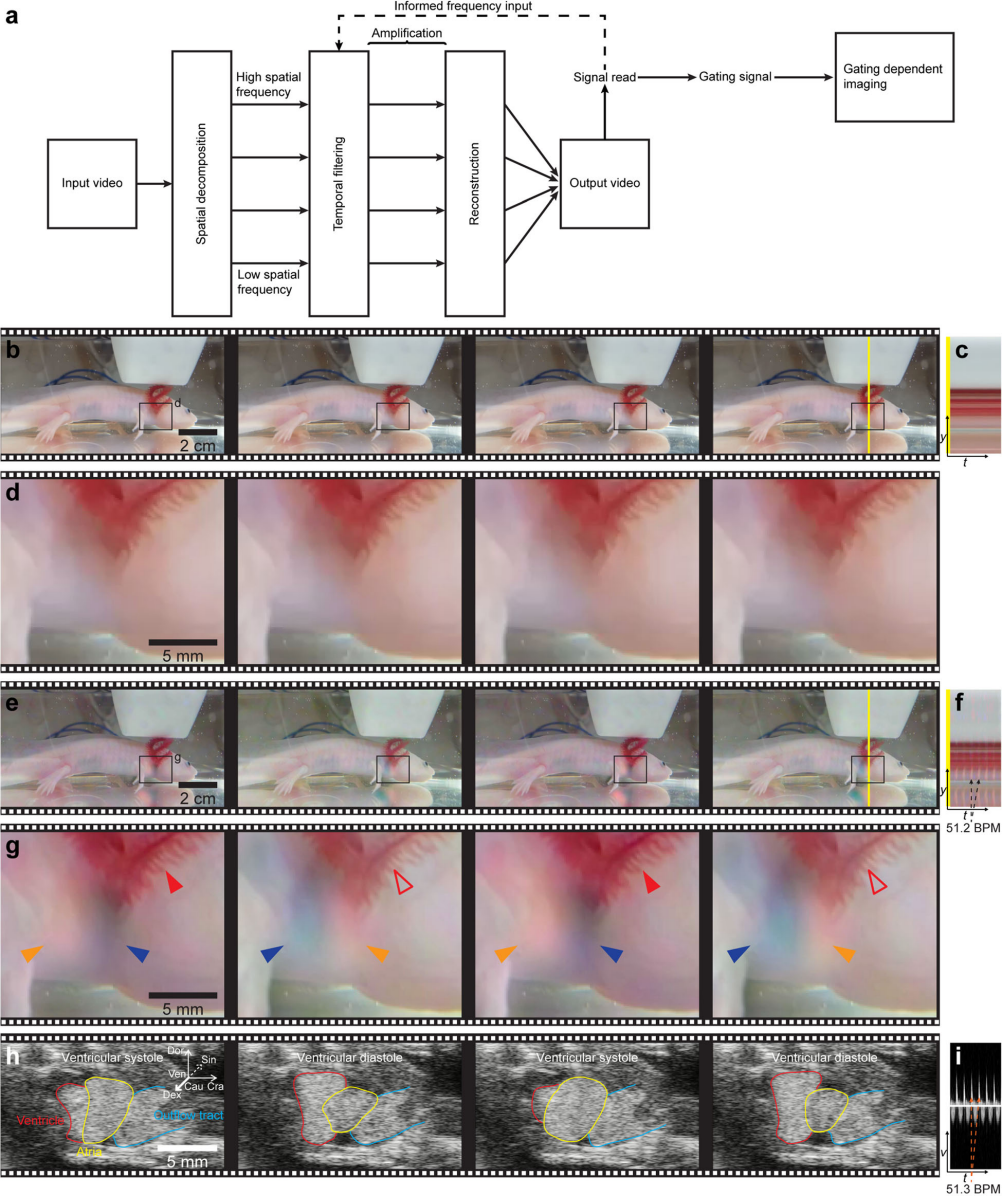
Overview and example of Eulerian video magnification procedure. **a** Block diagram representing the processing steps of a Eulerian Video Magnification procedure resulting in a signal read of the signal of interest or a gating signal for another imaging procedure. **b** Four frames from original input video source of anesthetized axolotl (Additional file 1, left trace). **c** Vertical scan line from the input source over time. **d** Magnification of box in **b** showing the cardiac region of the input video. No obvious color changes resulting from the beating heart. **e** The same four frames as in **b** with the axolotl’s pulse signal magnified. **f** Same scan line over time as in **c** but on color-magnified video. Color fluctuations are visible. **g** Magnification of box in **e**. Color changes are present over time in the cardiac region. **h** Four frames of brightness mode echocardiography recorded simultaneously with input video. The frames match the same time points as in **b**, **d**, **e**, **g**. **i** Pulsed-wave Doppler recording of blood flow in the ventricle. Note that peak flow matches lines in **f**, demonstrating that color changes in magnified video result from the beating heart

The world is teeming with invisible signals and information imperceptible to humans. Some animals have evolved sensory systems that allow them to take advantage of these, seemingly, insensible signals that fall outside the range of human perception. Examples of this are ultrasound perception in bats (visionless flight first described in [1], and ultrasound production/perception first described in [2, 3]) and cetaceans [4]; electroreception in a range of animals such as several bony fishes [5], elasmobranchs [6], and even mammals [7]; and magnetoreception in a range of migratory birds [8] and other species (reviewed in [9]). Recently, a novel technique named Eulerian video magnification (EVM) was developed to magnify minute color changes or motion, invisible to the naked eye, from videographic material [10]. In this technique, a video sequence is spatially decomposed (i.e., separated into different spatial frequency bands) and temporally filtered with an adjustable band-pass filter and the resulting signal is amplified to reveal hidden temporal variations (Fig. 1a. For full mathematical background, see [10]). The result of this procedure is a marked magnification of inherent local fluctuations in the video sequence, which can reveal the subtle skin color changes in the human face related to increased capillary perfusion after left ventricular ejection allowing for an easy heart rate (*f*_H_) measurement or render visible the faint motions related to breathing in infants [10]. A previous attempt has been made to extract human *f*_H_ from video material [11]; however, this procedure relies on sophisticated face-tracking algorithms and is unlikely to be applicable to other species. Likewise, other motion magnification techniques have been developed [12, 13]. However, these follow the Lagrangian perspective (with reference to fluid dynamics where the trajectory of particles is tracked over time, as opposed to the Eulerian perspective where the properties of a fluid evolve over time) and are subject to produce artifacts when applied to the complicated and unpredictable motions of animals. So far, EVM has not been used in any studies in the field of experimental biology, likely because the mathematical fundament of this technique is somewhat complicated and may be perceived by biologists as difficult to apply. However, the advantages of amplifying our sense of vision to perceive minute fluctuations with this tool should outweigh these concerns.

Being able to acquire a reliable and completely non-invasive measurement of *f*_H_ from video material may, arguably, be the most valuable application of EVM in experimental biology. Assuming that an animal is in metabolic steady-state in which energy is constantly replenished by aerobic metabolism, the metabolic rate (*MR*) and the rate of oxygen consumption [*V̇*(O_2_)] may be assumed to be equivalent. Fick’s convection equation for the cardiovascular system [14]:

1 $$ \dot{V} \left({\mathrm{O}}_2\right)={f}_{\mathrm{H}}\times {V}_{\mathrm{S}}\times \left[{C}_{\mathrm{a}}\left({\mathrm{O}}_2\right)-{C}_{\mathrm{v}}\left({\mathrm{O}}_2\right)\right] $$

where *V*_S_ is the stroke volume, *C*_a_(O_2_) is the O_2_ content of arterial blood, and *C*_v_(O_2_) is the O_2_ content of venous blood, summarizes the relationship between *f*_H_ and *V̇*(O_2_), and if the oxygen pulse {*V*_S_ × [*C*_a_(O_2_) − *C*_v_(O_2_)]} is constant or varies in a predictable way with changes in *f*_H_, then measurements of *f*_H_ can be used to evaluate *MR* of animals in both laboratory and field conditions [15]. The *f*_H_-*MR* relationship has been investigated in numerous studies on vertebrates of different classes under different circumstances, e.g., resting, walking, swimming, diving, flying, thermoregulating, and digesting (for review see [15]). Generally, a positive correlation between *f*_H_ and *MR* can be found in mammals, birds, and reptiles where *f*_H_ is the dominant component of changes in *V̇*(O_2_) [15]. In fishes (and presumably in amphibians, though this is not well-supported by literature), the general consensus has for a long time been that *V̇*(O_2_) is strongly regulated by the oxygen pulse (largely based on a classical study [16]), and therefore, a *f*_H_-*MR* relationship is more challenging to establish [17]. However, this view has recently been challenged, and it is now recognized that tachycardia at rest is likely to have biased many of the older studies of *f*_H_ in fish due to short recovery times after surgery and disturbances [18, 19].

Traditionally, externally attached or implantable biologging devices that record electrocardiogram or acoustic information are used to acquire *f*_H_ in the laboratory or in the wild. The advantage of this is continuous logging and the ability to monitor the animal in situations where the researcher cannot be present (extreme dives, flying, etc.). The disadvantage is that the device first has to be attached to, or implanted into, the animal which requires some degree of manual handling and often involves anesthesia. This may affect measurements for some time after perturbation and even continuously if the device affects the physiological or behavioral condition of the animal (e.g., [20]). Additionally, in most cases, these devices must be removed to recover the data, requiring a second manipulation or capture of the subject. Therefore, in some situations where filming may be applicable (either active filming or using passive “camera traps”), *f*_H_ obtained by EVM may be desirable. The temporal occurrence of color changes related to the propagating pulse wave associated with a heartbeat also carries information about pulse wave velocity. Additionally, other minute color changes or motions related to animal physiology or behavior such as breathing and sound production may be obtained by this technique.

In this study, we describe how EVM can be used to obtain inapparent signals, mostly focusing on *f*_H_. This is established through a series of laboratory-based experiments of heart rate detection in neotenic salamanders, fish, embryonic chickens, and mice, and finally with examples of captive (zoo) and free-ranging wildlife situations in which hidden signals can be magnified and detected.

## Results

First, we wanted to test if EVM would allow for *f*_H_ measurements under laboratory conditions where camera and light settings were easily adjustable. As the primary animal model for these initial steps, we selected the axolotl [*Ambystoma mexicanum* (Shaw and Nodder, 1798)], a Mexican caudate amphibian renowned for its tissue regenerative potential [21–23]. The axolotl is easily amenable to immersion-based anesthetics such as benzocaine (ethyl 4-aminobenzoate), MS-222 (ethyl 3-aminobenzoate methanesulfonic acid), and propofol (2,6-diisopropylphenol) [24] and does not require constant gill irrigation which reduces motion noise during video recording. The axolotl exists in different color strains, e.g., dark green/brown wild type, white leucistic, and albino, allowing for evaluation of the methodology on different color types. Additionally, the axolotl not only exhibits skin covered deep vascular beds but also very superficial vascular networks in their external gills which should result in areas with a high degree of color change during blood movement. To demonstrate the EVM method, we anesthetized a leucistic axolotl (body mass (BM) 10.73 g, total length (TL) 11.8 cm) in 200 mg/l benzocaine and video recorded (see Table 1 for video specifications) the water-covered and sedated animal from a lateral viewpoint, while simultaneously acquiring an echocardiographic signal for validation of *f*_H_ (Fig. 1). Although the original video recording did not display any obvious color changes or motions related to the animal’s heartbeat (Fig. 1b–d; Additional file 1, left trace), EVM-based color magnification (Fig. 1e–g; Additional file 1, right trace, first part) and motion magnification (Additional file 1, right trace, second part) both revealed rhythmic color changes and motions on the skin surface directly adjacent to the position of the heart and on the external gills, changing with the same frequency as the heartbeat signal acquired with ultrasonographic imaging (Fig. 1h–i; Additional file 1, third part and sound trace for all three parts).

**Table 1:** Specifications for included videos and Eulerian video magnification parameters

Related figure of video material	Number of videos	Spatial resolution (px^2^)	Temporal resolution (FPS)	Magnification type (C or M)	Initial EVM band-pass filter (Hz)	Informed EVM band-pass filter (Hz)	Channel (Com., R, G, B)	Magnification level
Figure 1b–g	1	1920 × 1080	30	C and M	0.1–3.0	0.8–0.9	All, Com.	100
Figure 2a–j	1	1920 × 1080	30	C	0.5–1.2	0.6–0.8	All, Com., G	100
Figure 2k–x	18	1920 × 1080	30	C	0.2–1.2	0.2–0.5 (rest), 0.6–0.8 (exercise and anesthesia)	All, G	100
Figure 3	6	3840 × 2160, 2560 × 1440, 1920 × 1080, 1920 × 1080, 1280 × 720, 640 × 480	30, 30, 60, 30, 30, 30	C	0.5–1.2	0.6–0.8	All, G	100
Figure 4a–c	1	1920 × 1080	30	M	0.2–0.8	0.3–0.5	All, G	10,000
Figure 4d–k	1	1920 × 1080	30	C	0.2–1.2	0.3–0.5	All, G	100
Figure 5	4	1920 × 1080	30, 60	C	1.8–3.0	1.8–2.4	All, B	50
Figure 6a, b	1	1280 × 720	120	C	0.5–1.2	0.8–0.9	All, Com.	100
Figure 6d–h	1	1280 × 720	120	C	0.9–1.3	1.0–1.1	All, Com.	100
Figure 6i–m	1	1280 × 720	120	C	0.9–1.3	1.0–1.1	All, Com.	100
Figure 7a–c	1	1920 × 1080	30	M	1.8–2.5	1.9–2.3	All, B	100
Figure 7d–g	1	1280 × 720	30	C	0.5–0.8	0.7–0.8	All, G	100
Figure 8a–n	156	1280 × 720	120	C	1.3–2.9 (day 1–3), 1.5–3.2 (day 3.5–5.0), 2.7–4.8 (day 6.0–9.0)	1.5–2.8 (day 1–3), 1.5–3.2 (day 3.5–5.0), 3.2–4.8 (day 6.0–9.0)	All, Com.	100
Figure 8o–v	1	1920 × 1080	30	C	1.0–2.2	1.5–1.9	All, Com.	100
Figure 10	4	1920 × 1080	50, 60	M	0–30	0–30	All, Com.	300
Figure 11	1	1920 × 1080	50	M	0–25	0–25	All, Com.	500

“Related figure of video material” specifies what figure parts the video has been used for. “Channel” specifies which channel of magnified videos that were analyzed for the initial magnification (for all video material, this was done for all channels including the combined channel signal) and for the subsequent informed processing

*px* pixel, *FPS* frames per second, *Com.* combined, *R* red, *G* green, *B* blue


**Additional file 1.** Simultaneous video recording for Eulerian video magnification and echocardiography. First two parts are dual trace videos of anesthetized leucistic axolotl. Original video to the left and Eulerian Video Magnified video to the right. First part shows color magnification, second part shows motion magnification. Third part is brightness mode echocardiographic video recorded simultaneously to filming. Note that all parts have a sound trace originating from pulsed wave Doppler ultrasound recorded simultaneously, revealing a match in heart rate with color changes and motion in the thoracic and gill region. Video relates to Fig. 1b-i.


### Optimization of EVM and physiological measurements

**Fig. 2:**
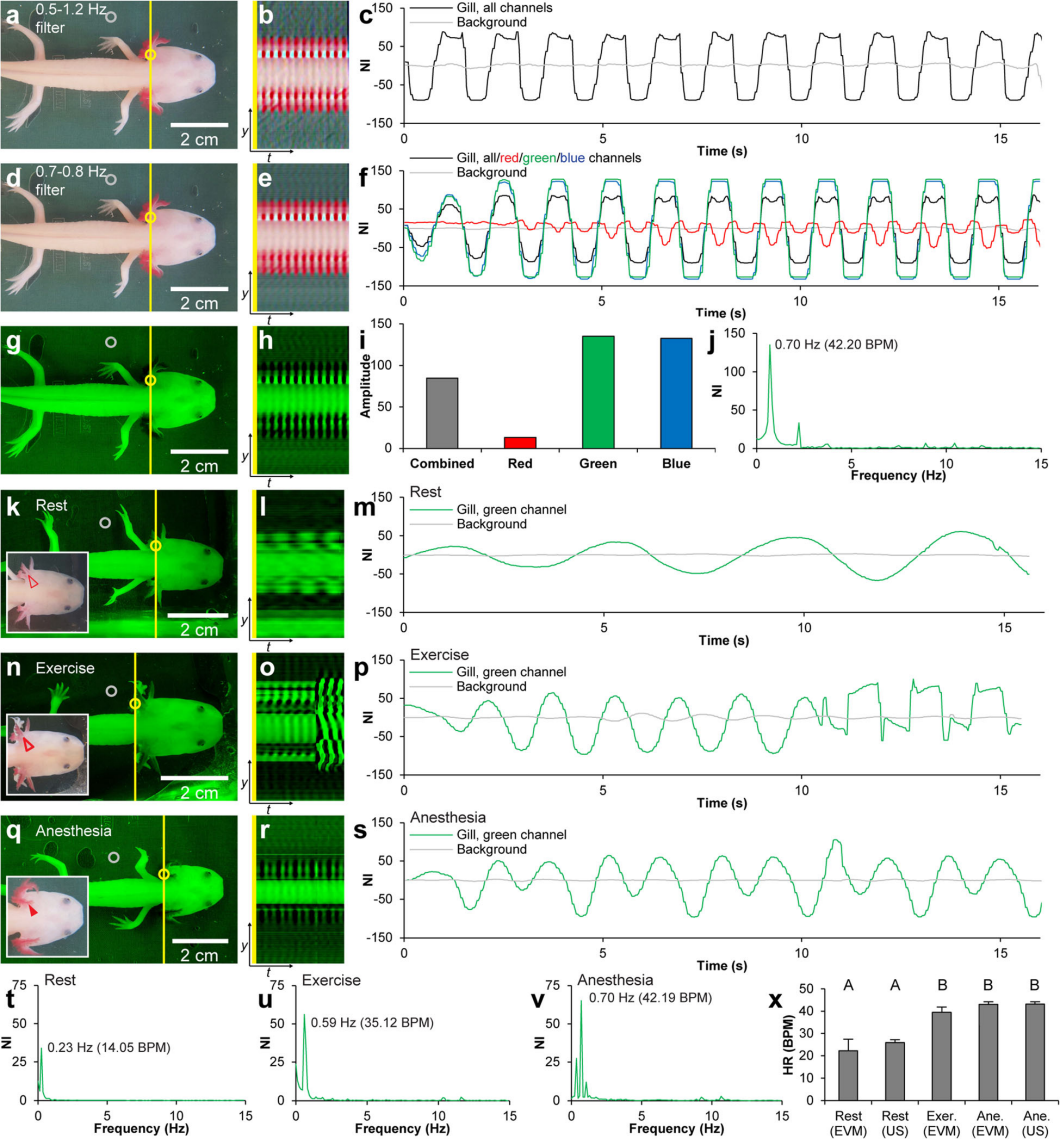
Band-pass filter and channel optimization and simple physiology experiment. **a** Single frame from color-magnified video of anesthetized axolotl applying a wide band-pass filter (0.5–1.2 Hz) (Additional file 2, first part). **b** Vertical scan line from magnified video over time. **c** Gill and background signal (yellow and gray rings in **a**) development over time. **d**–**f** Single frame (**d**), vertical scan line (**e**), and gill and background signal development over time (**f**) applying a narrow band-pass filter (0.7–0.8 Hz) (Additional file 2, second part). Notice less background noise in **e** than in **b**. Both the combined, red, green, and blue channel signals are displayed in **f** showing noticeable differences in signal amplitudes of the different channels. **g** Same frame as in **d** showing only the green channel. **h** Same vertical scan line as in **e** for the green channel. **i** Average signal amplitudes for the combined, red, green, and blue channel in the narrow band-pass filter magnified video. For this video, the green channel has the highest signal amplitude. **j** Fourier-transformed green channel signal in **f** in the frequency domain revealing an average heart frequency of 0.70 Hz (42.20 BPS). **k**–**m** Single frame (**k**), vertical scan line (**l**), and gill and background signal development over time (**m**) in resting axolotl (Additional file 3, first part). **n**–**p** Single frame (**n**), vertical scan line (**o**), and gill and background signal development over time (**p**) in exercised axolotl (Additional file 3, second part). **q**–**s** Single frame (**q**), vertical scan line (**r**), and gill and background signal development over time (**s**) in anesthetized axolotl (Additional file 3, third part). **t**–**v** Green channel signals from **m**, **p**, **s** in the frequency domain. **x** Bar chart (mean ± 95% confidence interval) of heart rate in rest, exercise, and anesthesia measured with Eulerian video magnification (EVM) and ultrasound (US) respectively. Letters (A and B) indicate statistically significant differences between groups


Next, we wanted to test if heartbeat signal detection could be optimized by applying a two-stage frequency-informed EVM procedure [Fig. 1a, i.e., first allow for a wide passband to pick up a heartbeat signal with an unknown frequency and then reprocess the video with a much narrower band-pass filter centered on the measured *f*_H_ to optimize signal to noise ratio (SNR)], and by selecting a specific channel (red, green, or blue) of the magnified video with standard 8-bit RGB colors. Additionally, we wanted to demonstrate a simple cardiovascular physiology relevant setup in which EVM-acquired *f*_H_ could be obtained for resting, exercised, and anesthetized axolotls. We studied six leucistic axolotls (BM 9.59 ± 1.68 g, TL 11.52 ± 0.63 cm) that were video recorded from a dorsal viewpoint after 48 h of fasting and 24 h of undisturbed rest (see Table 1 for video specifications), then gently subjected to echocardiographic *f*_H_ validation by submerging a remotely operated ultrasound transducer to a level 2 cm above the thoracic area of the animal. Thereafter the axolotls were exercised in a swimming respirometer to maximal swimming capacity by gradually increasing the water current until the moment when the animal was not able to produce sufficient force to maintain position against the water current. Video recordings were made directly after exercise. Echocardiographic *f*_H_ validation was not possible at this stage as the exercised axolotls were so agitated that positioning of the transducer in the required distance (< 2 cm) from the thoracic area resulted in escape behavior. Following an additional 24 h of undisturbed rest, benzocaine was added to the aquaria via catheters to induce anesthesia. After full anesthesia was reached (60 min [24]), video recordings were made of the sedated axolotls and these were subsequently subjected to echocardiographic *f*_H_ validation as described above. Again, EVM revealed a rhythmic color change signal in the gill region of all animals (exemplified in Fig. 2a–c). Applying a two-stage frequency-informed EVM procedure generated magnified videos with less noise (Fig. 2d, e, compare Fig. 2b with Fig. 2e; Additional file 2). Channel splitting revealed marked differences in signal amplitude in the red, green, and blue channels (Fig. 2f). Under the specific experimental light setting and the color pattern of the leucistic axolotls, the green channel displayed the largest signal amplitude (exemplified in Fig. 2f–j). In all subsequent experiments, the two-stage frequency-informed EVM and the channel selection procedure was incorporated to optimize band-pass filter and select optimal channel mode (red, green, blue, or all channels combined).

**Additional file 2** Effect of band-pass filter size on Eulerian video magnified material. Eulerian Video Magnified (color) recordings of anesthetized leucistic axolotl processed with a wide band-pass filter (first part) and a narrow band-pass filter (second part). Note the pronounced noise reduction when processing with a narrow band-pass. Video relates to Fig. 2a-f.


Benzocaine has a well-known tachycardiac effect in axolotls [24]; therefore, it was not surprising that both exercise and benzocaine-induced anesthesia resulted in increases in *f*_H_ (Fig. 2k–x; Additional file 3) and gill filament dilation (see inserts in Fig. 2k, n, q). A one-sample *t* test showed that the difference between *f*_H_ acquired by EVM and echocardiography in both resting and anesthetized conditions was not statistically significantly different from 0 (*p* = 0.30), thereby validating the accuracy of the EVM method. One-way ANOVA showed that EVM-acquired *f*_H_ differed statistically significantly between conditions (rest, exercise, and anesthesia) [F (2, 15) = 41.88, *p* < 0.01]. A Tukey Honestly Significant Difference post hoc test revealed that resting *f*_H_ was statistically lower than *f*_H_ following exercise and during anesthesia [Fig. 2x. Mean *f*_H_ (rest, EVM) ± 95% confidence interval (CI) = 22.23 ± 5.18 beats per minute (BPM), mean *f*_H_ (exercise, EVM) ± 95% CI = 39.44 ± 2.38 BPM, mean *f*_H_ (anesthesia, EVM) ± 95% CI = 42.99 ± 1.19 BPM. *f*_H_ (rest, EVM) vs. *f*_H_ (exercise, EVM): *p* < 0.001, *f*_H_ (rest, EVM) vs. *f*_H_ (anesthesia, EVM): *p* < 0.001], but *f*_H_ levels at exercise and anesthesia were not statistically significantly different from each other [*f*_H_ (exercise, EVM) vs. *f*_H_ (anesthesia, EVM): *p* = 0.33].


**Additional file 3.** Heartbeat magnification under three different physiological conditions using Eulerian video magnification. Eulerian Video Magnified (color) recordings of one leucistic axolotl at different physiological conditions. First part at rest, second part after exercise, third part under anesthesia. Note the frequency difference of color changes at the gills i.e. difference in heart rate under the different conditions. Video relates to Fig. 2k-x.


**Fig. 3:**
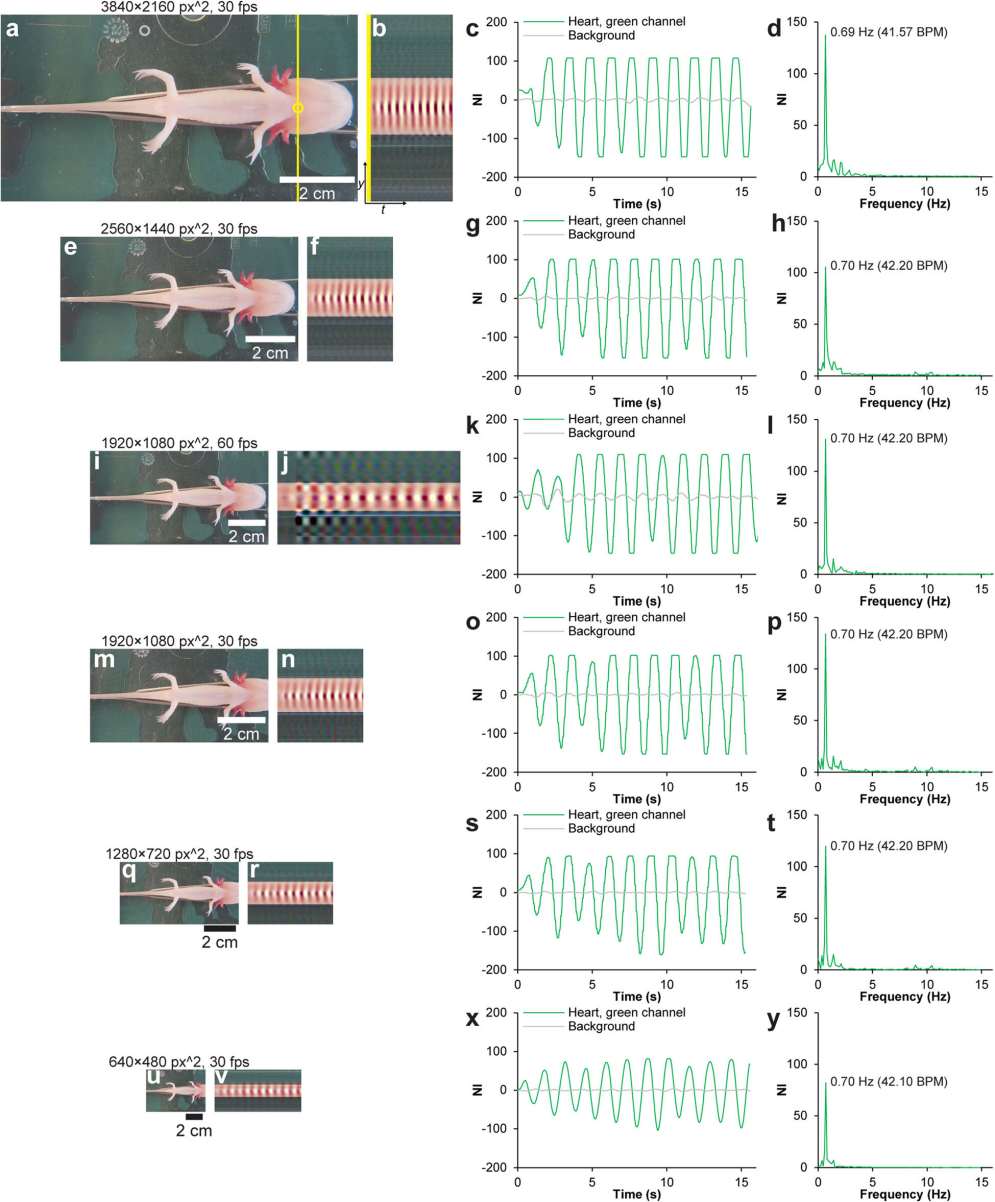
Applicability of videos with varying spatiotemporal resolution for Eulerian video magnification. **a** Single frame from color-magnified video with a spatiotemporal resolution of 3840 × 2160 px^2^, 30 fps of anesthetized axolotl (Additional file 4, first part). **b** Vertical scan line from magnified video over time. **c** Heart and background signal (yellow and gray rings in **a**) development over time. **d** Fourier-transformed green channel signal in **c** in the frequency domain. **e**–**h** Single frame (**e**), vertical scan line (**f**), gill and background signal development over time (**g**), and green channel signal in the frequency domain (**h**) in video with a spatiotemporal resolution of 2560 × 1440 px^2^, 30 fps (Additional file 4, second part). **i**–**l** Single frame (**i**), vertical scan line (**j**), gill and background signal development over time (**k**), and green channel signal in the frequency domain (**l**) in video with a spatiotemporal resolution of 1920 × 1080 px^2^, 60 fps (Additional file 4, third part). **m**–**p** Single frame (**m**), vertical scan line (**n**), gill and background signal development over time (**o**), and green channel signal in the frequency domain (**p**) in video with a spatiotemporal resolution of 1920 × 1080 px^2^, 30 fps (Additional file 4, fourth part). **q**–**t** Single frame (**q**), vertical scan line (**r**), gill and background signal development over time (**s**), and green channel signal in the frequency domain (**t**) in video with a spatiotemporal resolution of 1280 × 720 px^2^, 30 fps (Additional file 4, fifth part). **u**–**y** Single frame (**u**), vertical scan line (**v**), gill and background signal development over time (**x**), and green channel signal in the frequency domain (**y**) in video with a spatiotemporal resolution of 640 × 480 px^2^, 30 fps (Additional file 4, sixth part). Video frames are scaled relatively in spatial size to each other. All applied video formats allowed for heart rate detection on the anesthetized axolotl


Next, we wanted to test the importance of spatial and temporal resolution of video material intended for the EVM procedure since this has major implication on video file size and manageability. We recorded the same scene, an anesthetized leucistic axolotl positioned in a supine position exposing the thoracic region and the underlying beating heart, using six standard video frame formats: ultra-high definition [3840 × 2160 pixels (px)^2^, 30 frames per second (FPS)], quad-high definition (2560 × 1440 px^2^, 30 FPS), full-high definition with high speed (1920 × 1080 px^2^, 60 FPS), full-high definition (1920 × 1080 px^2^, 30 FPS), high definition (1280 × 720 px^2^, 30 FPS), and video graphics array (640 × 480 px^2^, 30 FPS). Subsequent to EVM, all video formats allowed for a clear heartbeat signal detection and *f*_H_ measurement (Fig. 3; Additional file 4).

**Additional file 4.** Effect of spatiotemporal resolution on Eulerian video magnified material. Eulerian Video Magnified (color) recordings of anesthetized leucistic axolotl at six different spatiotemporal resolutions. All resolutions allowed for heartbeat signal magnification and rate detection. Video relates to Fig. 3.


### Eulerian video magnification under non-optimal conditions

**Fig. 4:**
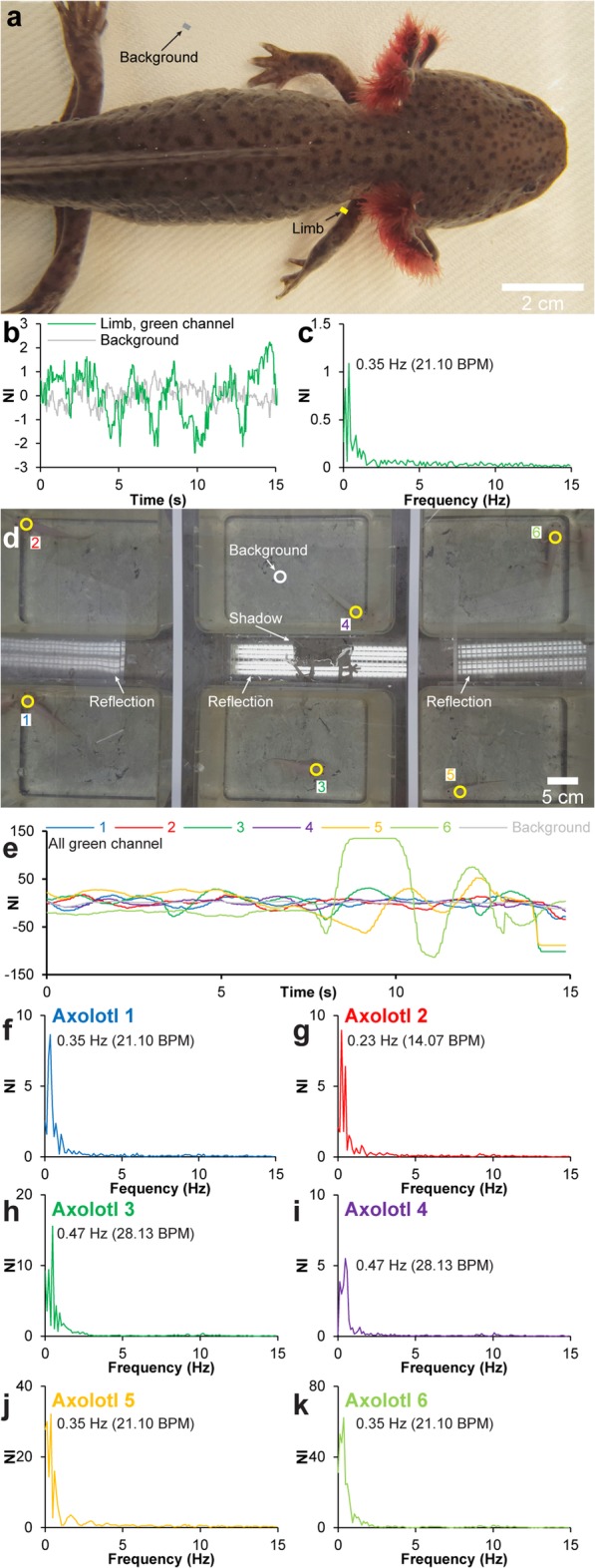
Heart rate detection in colored animal and in multi-animal setup. **a** Single frame from motion magnified video of brown wild-type axolotl (Additional file 5). **b** Proximal limb and background signal (yellow and gray lines in **a**) development over time (green channel). **c** Fourier-transformed green channel signal in **b** in the frequency domain. Although the enhanced motion signal related to the heart beat of the colored animal is less pronounced relatively to the color change signal in leucistic animals (see Figs. 1, 2 and 3), a heart rate measurement is still measureable. **d** Single frame from color-magnified video of a multi-animal setup containing six resting axolotls (Additional file 6). **e** Cervical and background signal (yellow and white rings in **d**) development over time of each of the six axolotls in **d** (green channel). **f**-**k** Fourier-transformed green channel signals in **e** in the frequency domain. Resting heart rate varied between 14.07 and 28.13 BPM

In colored axolotls, subtle color changes in the thoracic region are not as pronounced as in leucistic and more translucent individuals, which likely applies to other species as well. However, our initial test on the axolotl revealed that even small rhythmic motions in the front limbs related to blood movement in the brachial arteries could be detected with EVM-based motion magnification (Additional file 1). Therefore, we wanted to test the applicability of EVM-based motion magnification to detect *f*_H_ via movements of the front limb in a dark brown wild-type axolotl. A large wild-type axolotl (BM 61.90 g, TL 19.7 cm) was anesthetized in 200 mg/l benzocaine, video recorded (for video specifications see Table 1), and the video was subsequently processed for motion magnification. By placing the image probe for motion detection directly on the interface of the upper limb and background in the magnified video, heart rate detection was possible (Fig. 4a–c; Additional file 5).

To mimic an experimental setup in which *f*_H_ needs to be monitored in multiple animals at the same time with a limited number of cameras and with non-optimal light and background settings, we used the same six leucistic animals as in the rest/exercise/anesthesia experiment. The six axolotls were housed in individual plastic aquaria and placed on a gray linoleum background. Following 48 h of fasting and 24 h of undisturbed rest, a video recording containing all six animals was performed (see Table 1 for video specifications). Although axolotls are relatively sedentary animals, the chances of encountering movements during recording increases with the number of animals being recorded at the same time and the duration of recording. Figure 4d and Additional file 6 display a recording in which five out of six animals did not move during the 15-s recording, whereas animal 6 did move for the last 5 s. In spite of little contrast between axolotls and background, and pronounced reflections and shadows in the scene (Fig. 4d), it was still possible to acquire *f*_H_ for all six animals [mean *f*_H_ (rest, EVM) ± 95% CI = 22.27 ± 4.24 BPM; Fig. 4d–k] and a paired *t* test showed that resting heart rate was not significantly different from previous individual recordings of the same animals (*p* = 0.99).


**Additional file 6.** Heartbeat magnification in multi-animal setup using Eulerian video magnification. Eulerian Video Magnified (color) recording of six leucistic axolotls at rest. Video relates to Fig. 4d-k.


**Fig. 5:**
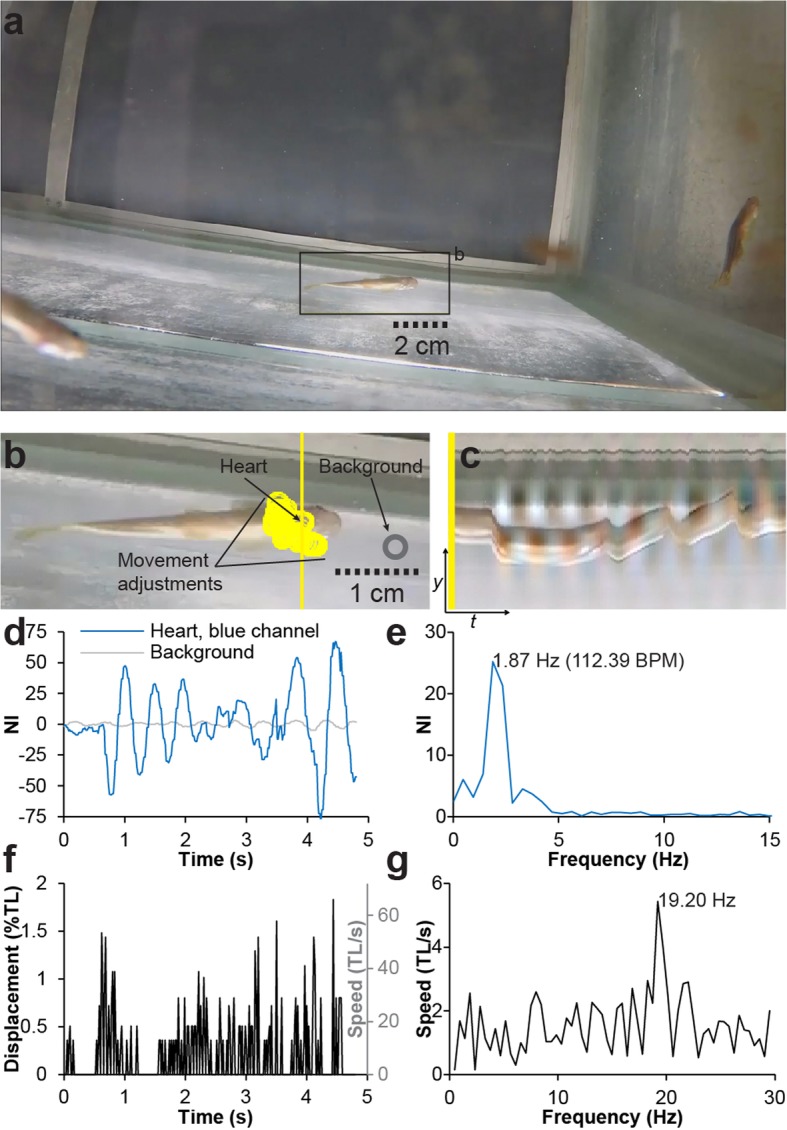
Heart rate detection in conscious, unrestrained, group-housed zebrafish. **a** Single frame from color-magnified video of wild-type conscious and unrestrained adult zebrafish (Additional file 7). **b** Magnification of box in **a** with a display of regions of interest (yellow rings) tracking the ventral surface of the thoracic region as the fish moves gently. **c** Vertical scan line from magnified video over time showing temporal variation of color at the level of the thoracic region. **d** Thoracic and background signal (yellow and gray rings in **b**) development over time (blue channel). **e** Fourier-transformed blue channel signal in **d** in the frequency domain. **f** Displacement in percent of fish total length (TL) (left vertical axis) and speed in TL/s (right vertical axis) of thoracic region of interest (yellow rings in **b**) during the recording. Although the fish movements are rapid and occur at a high rate, the displacement is small and the rate is out of the frequency range of the beating heart (compare **d** with **f**). **g** Fourier-transformed speed of displacement signal in **f** in the frequency domain. Scale bars in **a** and **b** are approximations (indicated by dotted lines) based on average zebrafish sizes in the batch since the fish was at an unknown depth in the water column during the recording


In order to test if EVM allowed for *f*_H_ detection in a more active ectotherm than the axolotl, we used conscious, unrestrained zebrafish [*Danio rerio* (Hamilton, 1822)]. Forty-five adult (> 4 months of age) mixed sex, wild-type zebrafish (representative group of 13 animals euthanized for parallel research had BM 457.15 ± 109.09 mg, TL 39.08 ± 2.02 mm) were housed in a 200-l aquarium with fully oxygenated water (90 l) at 27.6 °C. Following 36 h of fasting and 24 h of undisturbed rest to minimize post absorptive heart rate changes and disturbance, video recordings were performed with a remotely controlled camera (see Table 1 for video specifications). Although zebrafish are active animals, it was possible to acquire four videos of eight separate instances over a 15-min period in which the movements of the fish, although rapid (mean movement speed ± 95% CI = 9.36 ± 2.44 TL/s), were brief enough and out of the frequency range of expected *f*_H_ that EVM allowed for *f*_H_ extraction (Fig. 5; Additional file 7; mean *f*_H_ (EVM) ± 95% CI = 127.57 ± 7.87 BPM).

**Additional file 7.** Heartbeat magnification in conscious, unconstrained zebrafish using Eulerian video magnification. Eulerian Video Magnified (color) recording of wild type conscious and unconstrained adult zebrafish. Although the fish is moving slightly during the recording resulting in an increased level of noise particularly in the caudal part of the fish, a heartbeat signal can be extracted from the ventral thoracic surface. Video relates to Fig. 5.


### Pulse wave velocity measurement with EVM

**Fig. 6:**
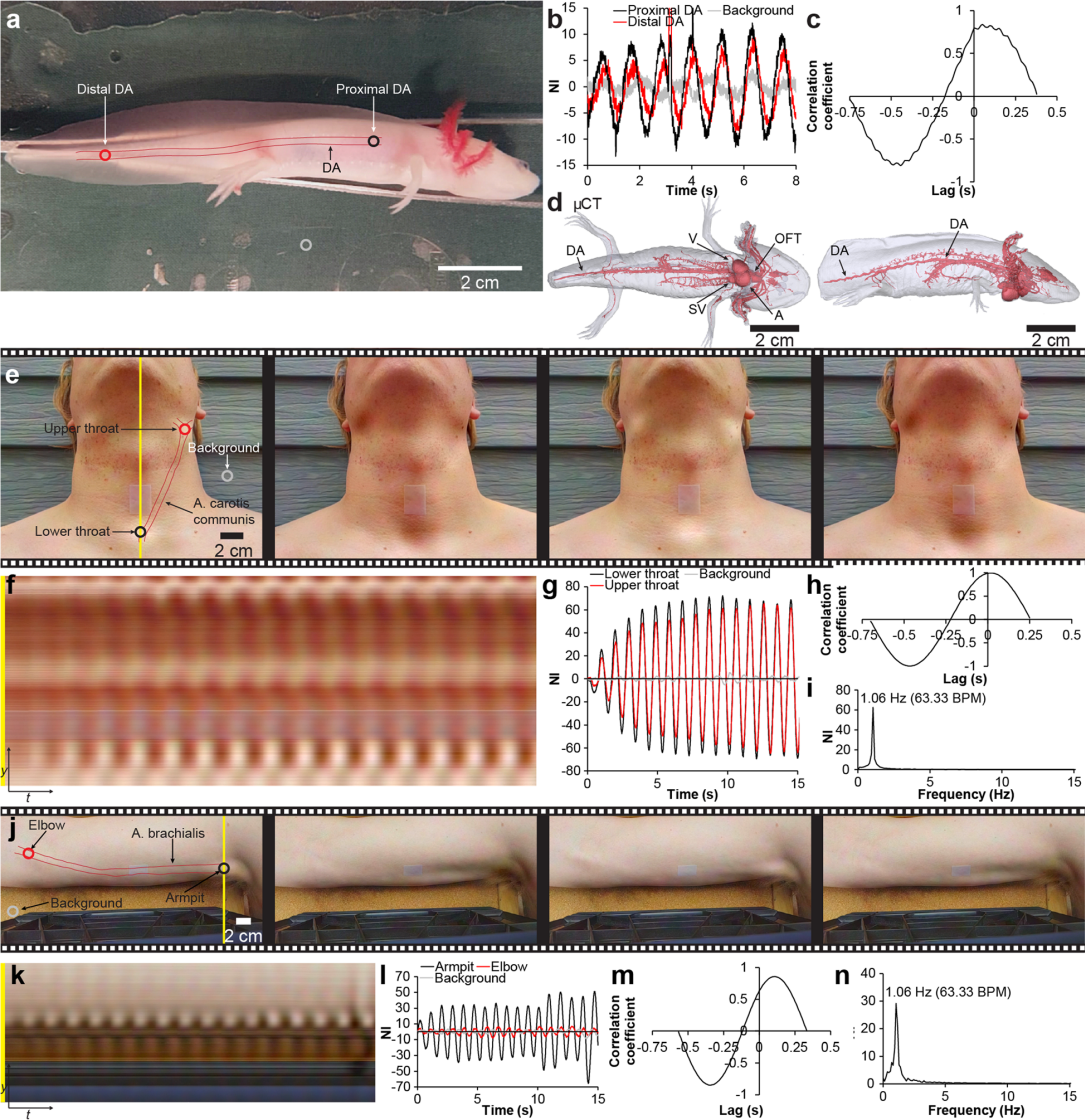
Pulse transit time detection in magnified recordings in animals with low and high blood pressure. **a** Single frame from color-magnified video of an anesthetized axolotl (Additional file 8). The dorsal aorta (DA) is indicated. **b** Proximal and distal DA and background signal (black, red and gray rings in **a**) development over time (combined channel signals). **c** Cross-correlation plot of proximal and distal DA signal. Notice the phase delay in the proximal and distal DA signal .**d** Micro-CT (μCT) generated model of the heart [sinus venosus (SV), atria (A), ventricle (V), and outflow tract (OFT)] and major arterial networks in the axolotl confirming the course of the DA as drawn in **a**. **e** Four-frame time series showing color changes along the left arteria carotis communis over time in a color-magnified video of a resting human being (Additional file 9, first part). **f** Vertical scan line over time showing temporal variation at the intersection with the a. carotis communis. **g** Lower and upper throat and background signal (black, red, and gray rings in **e**) development over time (combined channel signals). **h** Cross-correlation plot of lower and upper throat signal. Notice the phase delay between the lower throat and upper throat signal. **i** Fourier-transformed combined channel signal for lower throat in **g** in the frequency domain. **j** Four-frame time series showing color changes along the right arteria brachialis over time in a color-magnified video of a resting human being (Additional file 9, second part). **k** Vertical scan line over time showing temporal variation at the intersection with the a. brachialis. **l** Armpit and elbow and background signal (black, red, and gray rings in **j**) development over time (combined channel signals). **m** Cross-correlation plot of armpit and elbow signal. Notice the phase delay between the lower armpit and elbow signal. **n** Fourier-transformed combined channel signal for armpit in **i** in the frequency domain


Eulerian video magnification operates on a pixel basis; therefore, a time shift between two or more rhythmic temporal phenomena, such as a propagating pulse wave causing a directional color change, can in theory be detected at different locations given that the temporal resolution of the camera is sufficient to detect the time gap between the pulse arriving at the locations of interest. To test if EVM allowed for detection of pulse wave velocity (PWV), we first examined the low pressure, low PWV vascular system of the amphibian axolotl. An anesthetized leucistic axolotl (BM 11.30 g, TL 11.7 cm) was positioned on the side (Fig. 6a) and recorded at a high frame rate (120 FPS, see Table 1 for additional specifications). The recording was processed with EVM (Additional file 8), and color fluctuations were measured at two positions in the vascular system (Fig. 6b, c). The amphibian dorsal aorta (DA) originates from the efferent gill arteries and runs in the caudal direction along the long axis of the animal (Fig. 6d). A proximal DA and a distal DA position separated by 64.22 mm was selected, and the time gap between signal peaks and the pulse transit time (PTT) was measured as mean PTT = 71.22 ms resulting in a mean PWV = 0.90 m/s. To test the procedure on a high pressure, high PWV vascular system, we examined a resting male human being (*Homo sapiens* Linnaeus, 1758) [BM 95 kg, TL 184 cm, *f*_H_ (rest, EVM) = 63.33 BPM] and repeated the procedure described above on the throat (PWV in the left arteria carotis communis) (Fig. 6d–i; Additional file 9, first part), and the upper arm (PWV in the right arteria brachialis) (Fig. 6i–n; Additional file 9, second part). Time shift at the two most distant positions of the arteria carotis communis with detectable color fluctuations (separated by 96.13 mm) was close to the temporal detection limit (Fig. 6g, h) and resulted in a mean PTT = 21.95 ms and mean PWV = 4.38 m/s. The two upper arm locations (armpit and elbow separated by 264.08 mm) displayed an easily detectable phase shift (Fig. 6l, m) with a mean PTT = 72.86 ms and mean PWV = 3.62 m/s.

**Additional file 8.** Pulse wave transit time detection in low blood pressure animal using Eulerian video magnification. Eulerian Video Magnified (color) recording of one anesthetized leucistic axolotl. Pulse wave propagation along the dorsal aorta can be detected at different positions allowing for a pulse transit time measurement. Video relates to Fig. 6a-c.



**Additional file 9.** Pulse wave transit time detection in high blood pressure animal using Eulerian video magnification. Eulerian Video Magnified (color) recordings of resting human being. Dual trace video. Original video to the left and color magnified video to the right. First part shows color magnification on the throat demonstrating pulsations in the arteria carotis communis, second part shows color magnification on the upper arm demonstrating pulsations in the arteria brachialis. Pulse wave propagation along the arteries can be detected at different positions allowing for a pulse transit time measurement. Video relates to Fig. 6d-m.


### Eulerian video magnification of non-dedicated videographic material

**Fig. 7:**
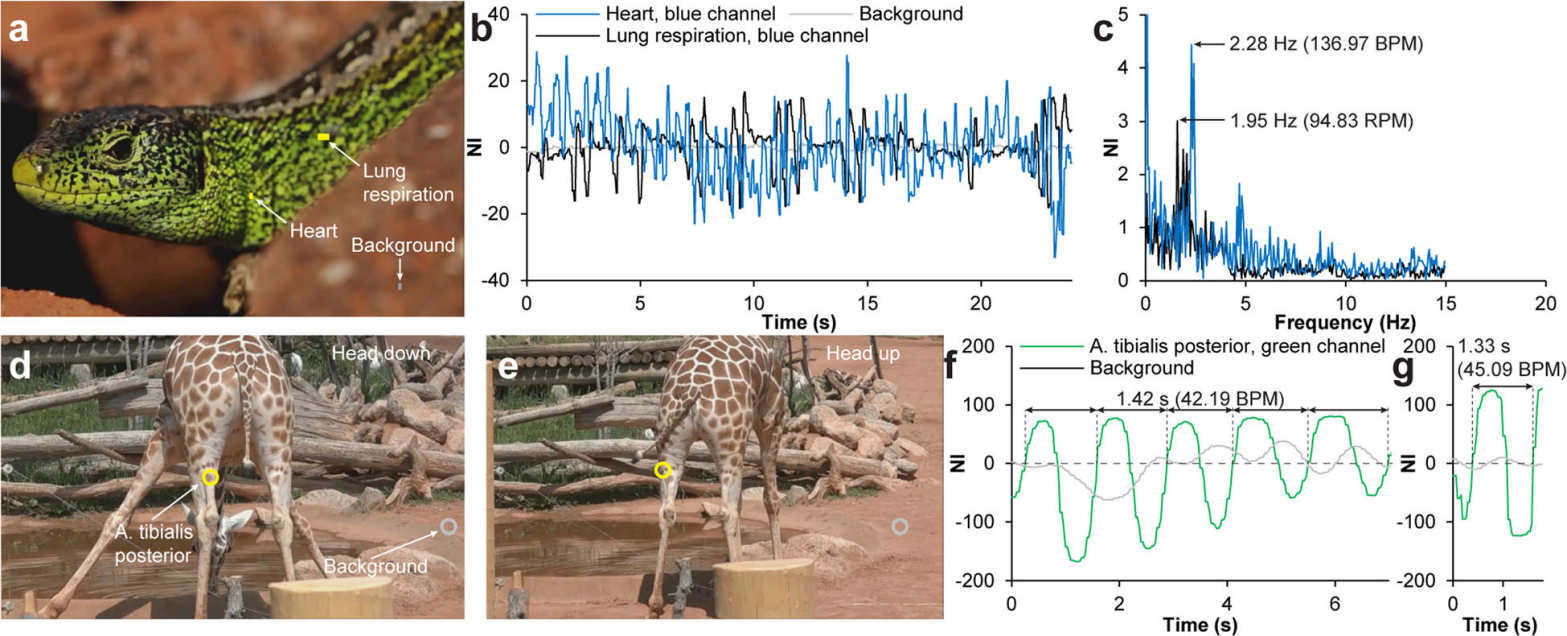
Signal detection with Eulerian video magnification in non-dedicated videographic material. **a** Single frame from motion magnified video of sand lizard. Video was located on Flickr and used with permission from Ben Locke. **b** Heart, lung, and background signal (yellow and gray lines in **a**) development over time (blue channel). **c** Fourier-transformed blue channel signal of heartbeat and respiration in **b** in the frequency domain. **d**, **e** Single-frame color-magnified video of a drinking giraffe (**d**) that eventually lift its head (**e**). Video was located on YouTube and used with permission from AliasAnimo. **f**, **g** Arteria tibialis posterior and background signal (yellow and gray rings in **d** and **e**) development over time (green channel). The low number of cardiac cycles in the timespan of the two postures does not allow for meaningful Fourier transformation; thus, heart rate is estimated as an average of the available signal periods for the two postures (5 periods during head down, one period during head up)

To test the applicability of EVM on animal videographic material that was not produced with the purpose of EVM, two online videos were located and analyzed with permission from the producers. The first video displayed a basking sand lizard (*Lacerta agilis* Linnaeus, 1758) (Fig. 7a). Following motion-enhanced EVM, mean *f*_H_ (136.97 BPM) and mean respiration rate (94.83 respirations per minute) could be measured over multiple cycles at the level of the heart and the lungs respectively (Fig. 7b, c). The second video displayed a drinking giraffe [*Giraffa camelopardalis* (Linnaeus, 1758)] that eventually lifted its head up (Fig. 7d-e). Here, color-enhanced EVM was used to the posterior part of the hind limb where the arteria tibialis posterior runs close to the surface and the fur layer is relatively thin. This allowed for *f*_H_ signal detection over five cardiac cycles while the head was down (mean *f*_H_ = 42.19 BPM) (Fig. 7f) and one cardiac cycle when the head was elevated (*f*_H_ = 45.09 BPM) (Fig. 7g).

### Embryonic heart rate detection and optical gating

**Fig. 8:**
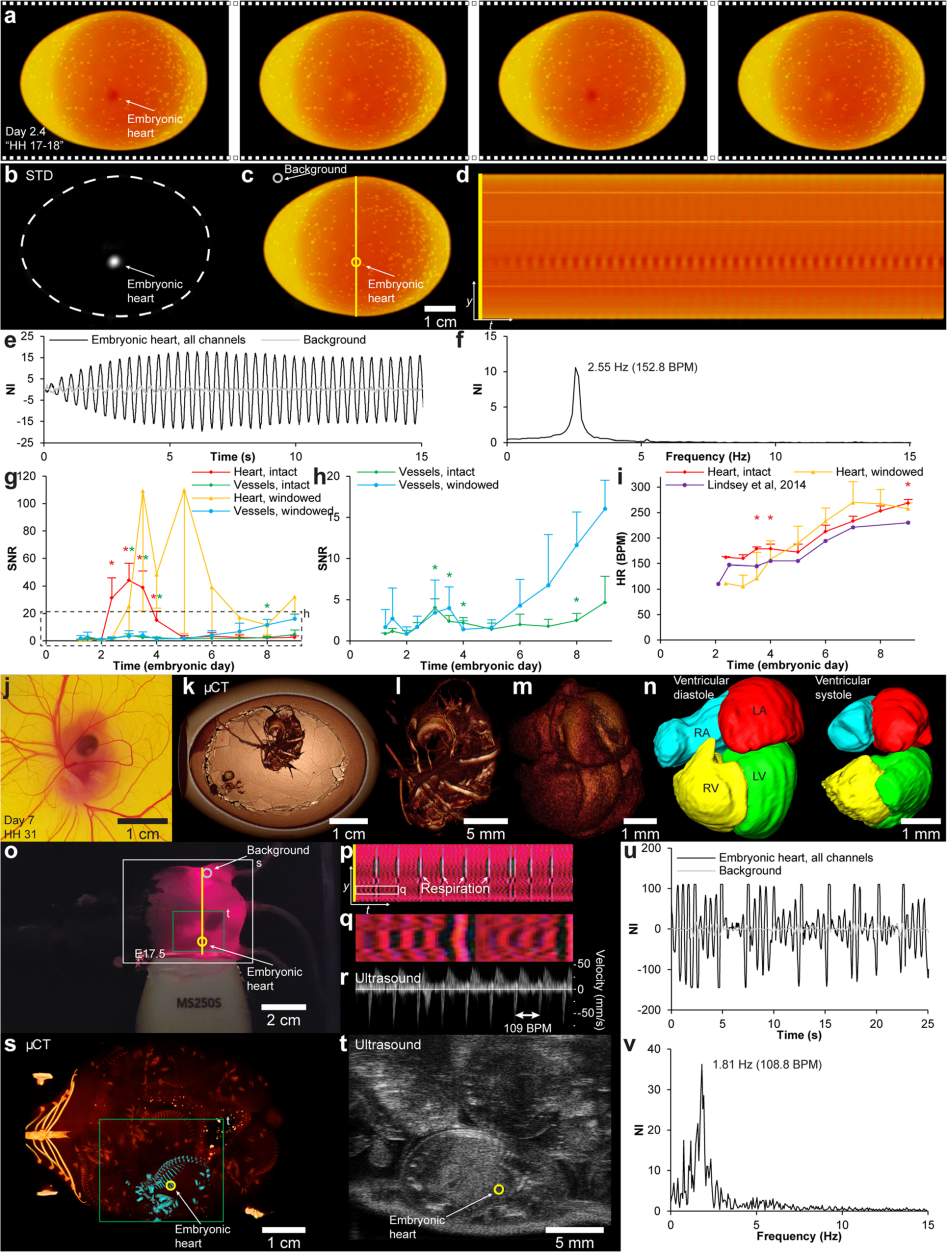
Heart rate detection and acquisition of gating signal in embryonic animals. **a** Four-frame time series showing color changes associated with the heartbeat in color-magnified video of embryonic chicken at day 2.4 in a candled, non-windowed egg (Additional file 10). **b** Pixel-wise standard deviation (STD) of magnified video highlighting embryonic heart. **c**, **d** Image probes (**c**) and vertical scan line over time (**d**) showing temporal variation at embryonic heart intersection. **e**, **f** Embryonic heart and background signal (yellow and gray rings in **c**) development over time (**e**) (combined channels), and Fourier-transformed signal in the frequency domain (**f**). **g** Line chart (mean ± 95% confidence interval) of heartbeat signal to noise ratio (SNR) during 9 days in intact and windowed eggs measured at the embryonic heart or vitelline vessels. **h** Enlargement of box in **g** (only vessel signals). Color coded asterisks (*) in **g**, **h** indicate statistically significant differences from SNR = 1. **i** Line chart comparing heart rate acquired with video magnification to reference values (Lindsey et al., 2014). Asterisks indicate statistically significant differences from reference values. **j** Embryonic chicken at day 7. **k**–**n** Three-dimensional rendering of embryonic chicken at day 7 from micro-CT (μCT) imaging gated by magnified optical signal. Volumetric reconstruction of the heart and vascular system of the embryo in a resealed egg (**k**–**m)** and segmentation of the heart chambers in two phases (**n**). **o** Single frame from color-magnified video of pregnant mouse abdomen (E17.5) (Additional file 12). **p** Vertical scan line from magnified video over time. **q** Magnification of box in **p**, showing color fluctuations associated with embryonic heartbeat. **r**–**t** Ultrasound (**r**, pulsed wave Doppler; **t**, brightness mode) and μCT (**s**) validation of heart rate and position of embryo. **u**, **v** Embryonic heart and background signal (yellow and gray rings in **o**) development over time (**u**) (combined channels), and Fourier-transformed signal in the frequency domain (**v**)


Development of embryonic domestic chickens [*Gallus gallus* (Linnaeus, 1758)] can be directly observed both by incubation in ovo with a window in the eggshell or in ex ovo cultures in which the eggshell is completely removed [25, 26]. The developing heart is visible at early stages of development and starts to contract after 29–45 h of development [Hamburger Hamilton stage (HH) 9–10] [27–30], allowing for an easy validative measurement of *f*_H_ under these incubation conditions. We wanted to test if EVM would allow for *f*_H_ detection through the intact eggshell during the first 9 days of embryonic development. A group of ten intact eggs and three windowed eggs (windowed at embryonic day 1.25) were video recorded (for video specifications see Table 1) at 12 time points during the first 9 days of embryonic development [embryonic day 1.25 (HH 6–7), 1.5 (HH 10–11), 2.0 (HH 12–13), 2.38 (HH 17–18), 3.0 (HH 19–20), 3.5 (HH 21–23), 4.0 (HH 24), 5.0 (HH 27), 6.0 (HH 29), 7.0 (HH 31), 8.0 (HH 34), 9.0 (HH 35). For intact eggs, the HH stage could only be assumed]. Video recordings were subsequently processed with EVM to enhance color changes associated with the heartbeat at the position of the heart and at the vitelline vessels (Fig. 8a–f, Additional file 10). One-way ANOVA showed that both heart and vitelline vessel SNR were statistically significantly different for different embryonic time points for both intact and windowed eggs [intact egg, heart: *F* (11, 108) = 21.40, *p* < 0.01; intact egg, vessels: *F* (11, 108) = 3.95, *p* < 0.01; windowed egg, heart: *F* (11, 24) = 3.01, *p* = 0.01; windowed egg, vessels: *F* (11, 24) = 8.27, *p* < 0.01]. Post hoc tests using the Bonferroni correction (*α* = 0.05/12 = 0.0042) showed that SNR was statistically significantly larger than 1 for the heart measurements on intact eggs at embryonic day 2.38 (*p* = 0.0014), 3.0 (*p* < 0.0008), 3.5 (p < 0.0008), and 4.0 (*p* = 0.0013) (Fig. 8g) and for the vitelline vessel measurements on intact eggs at embryonic day 3.0 (*p* < 0.0008), 3.5 (*p* = 0.0020), 4.0 (*p* = 0.0026), and 8.0 (*p* = 0.0038) (Fig. 8g, h). Signal to noise ratio for heart and vessel signals displayed a high degree of variability (Fig. 8g, large 95% CI error bars), which, based on our observations during the experiment, were likely caused by individual orientation of the embryo relative to the eggshell and variance in eggshell thickness, causing some embryos to produce very low SNR for heart and vitelline vessel signal. For all time points from embryonic day 5.0–9.0, there was at least one embryo in the intact eggshell group that displayed an EVM enhanced heart or vitelline vessel signal with a SNR > 6 (for the windowed egg group at least one embryo per time point in the same time span displayed a heart or vitelline vessel signal with a SNR > 17). Mean *f*_H_ was calculated for all time points (Fig. 8i) and a two-way mixed ANOVA showed that there was no statistically significant difference in *f*_H_ between the intact eggshell and windowed groups [*F* (1, 4) = 0.84, *p* = 0.41], but there was a statistically significant different in *f*_H_ between different embryonic time points [*F* (8, 32) = 152.56, *p* < 0.01]. Six of the selected time points matched embryonic days for which *f*_H_ has previously been reported (Lindsey et al., 2014). Bonferroni-corrected (*α* = 0.05/6 = 0.0083) *t* tests showed that *f*_H_ for embryos in intact eggs were statistically significantly different from reference values at embryonic day 3.5 (*p* < 0.002), 4.0 (*p* < 0.002), and 9.0 (*p* < 0.002).

**Additional file 10.** Heartbeat magnification in embryonic chicken using Eulerian video magnification. Eulerian Video Magnified (color) recording of fertilized chicken egg at day 2.4 of embryonic development. The embryonic heartbeat can be detected through the eggshell of the candled egg. Video relates to Fig. 8a-f.


With the knowledge of some embryos/eggs being predisposed as good candidates for EVM, we went on to test if real-time EVM could be used to enhance the heart beat signal through the eggshell and allow for optical gating of an in vivo micro-CT system to acquire volumetric data of the beating heart in selected cardiac phases. Eggs were carefully windowed at embryonic day 3.5, and the excised cap was maintained for later repositioning. Three embryos with an appropriate orientation that qualified them as good EVM candidates were microinjected in a vitelline vein with 100 μl of an in vivo vascular contrast agent (Exitron nano 12000, Miltenyi Biotec, Bergisch Gladbach, Germany) on embryonic day 7.0 (see Fig. 8j for embryo size). The excised eggshell cap was carefully repositioned, and the air phase between egg yolk surface with the floating embryo and the eggshell cap was replaced with Tris-buffered isotonic saline to bring the embryo in close proximity to the eggshell, as in the natural non-windowed condition. The egg was positioned on the in vivo micro-CT scanner bed, candled and heated with a flashlight, and recorded with an endoscope camera. Real-time EVM was used to acquire the color-enhanced heart beat signal, and using a custom made software, a gating signal was fed to the micro-CT system to perform a prospectively gated acquisition of projections with the heart in the same cardiac phase. Two acquisitions were made to capture volumetric data of the heart in the end-diastole and the end-systole phases (Fig. 8k–n, Additional file 11). Stereological examination of left and right ventricle blood volume in end-diastole and end-systole yielded a mean stroke volume (SV) of SV (left ventricle) ± 95% CI = 1.57 ± 0.28 mm^3^ and SV (right ventricle) ± 95% CI = 2.40 ± 1.34 mm^3^ and mean ejection fractions of EF (left ventricle) ± 95% CI = 47.17 ± 6.08% and EF (right ventricle) ± 95% CI = 63.35 ± 11.62%.

In contrast to the embryonic bird, the embryonic mammal is more difficult to access and observe. To test if a heartbeat signal could be acquired from an embryonic mammal in utero, we examined a pregnant C57BL/6 mouse with 10 embryos (number of embryos validated by post-mortem micro-CT imaging and dissection) at embryonic day 17.5 (E17.5). After induction of anesthesia, the adult mouse had abdominal hair removed and was placed in a black box with a cold light source providing trans-illumination (Fig. 8o). The mouse was video recorded (see Table 1 for video specifications) for subsequent EVM processing (color enhanced) while ultrasound imaging was simultaneously used to validate *f*_H_ (Additional file 12). Four embryos were positioned in such a way that *f*_H_ measurements were possible with both EVM (mean *f*_H_ ± 95% CI = 97.40 ± 8.13 BPM) and ultrasound (mean *f*_H_ ± 95% CI = 95.5 ± 8.09 BPM) (measurements shown for one embryo in Fig. 8p–v) which was not statistically significantly different (*p* = 0.52) when tested with a paired *t* test.


**Additional file 12.** Heartbeat magnification in embryonic mouse using Eulerian video magnification. Eulerian Video Magnified (color) recording of pregnant mouse with 10 embryos at day 17.5 of embryonic development. The embryonic heartbeat of some embryos can be detected in utero of the candled mother. Video relates to Fig. 8o-v.


### Infrasound detection

**Fig. 9:**
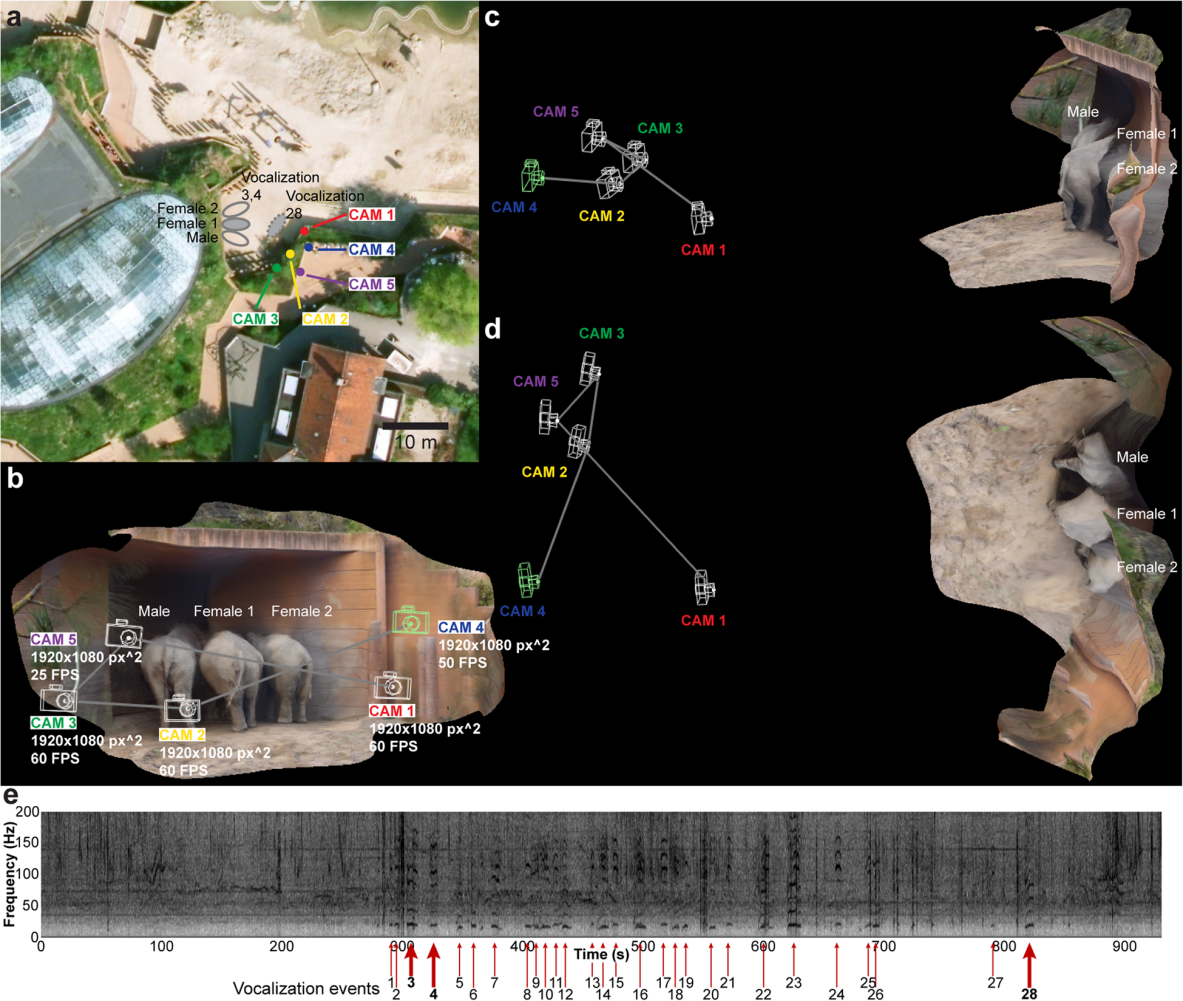
Multi-camera recording of vocalizing Asian elephants. Overview of scene. **a** Aerial photography of elephant enclosure in Copenhagen Zoo and the position of cameras (CAM 1–5) and elephants during video recordings. Used with permission from COWI. **b**, **c** Three-dimensional reconstructed scene viewed from behind the cameras (**b**), from the side (**c**), and from the top (**d**). **e** Audio spectrogram of the full duration of the video capture from the microphone in CAM 2. The 28 discrete vocalization events during the video capture are highlighted. Vocalization events 3, 4, and 28 were used in subsequent analyses of animal vibration. Position of relevant elephants during these events are displayed in **a**

**Fig. 10:**
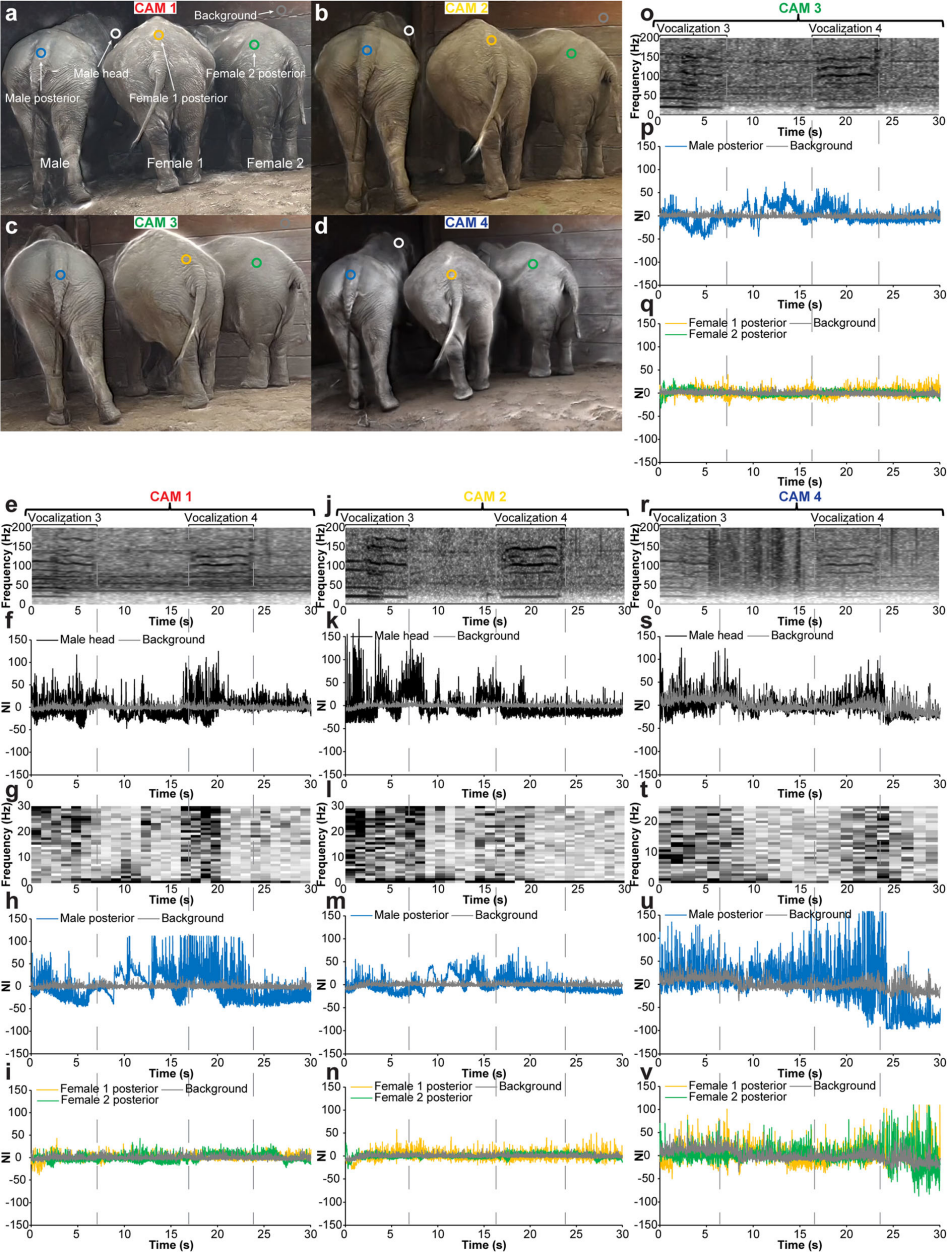
Multi-camera recording of three Asian elephants. Determination of vocalizing elephant with Eulerian video magnification. **a**–**d** Corresponding images from video recordings from CAM 1 (**a**), CAM 2 (**b**), CAM 3 (**c**), and CAM 4 (**d**). Image probes for vibration analysis are indicated on images and labeled in **a**. **e**–**v** Analysis of vibrations at different body positions of the three elephants during the video capture. The audio spectrograms associated with each recording are displayed at the top (**e**, **j**, **o**, and **r**), and the durations of the two vocalization events (events 3 and 4 in Fig. 9) are highlighted with dashed lines. Plots of normalized pixel intensity (NI) over time for image probes placed on the male head (**f**, **k**, and **s**), the male posterior (**h**, **m**, **p**, and **u**), the female 1 and female 2 posterior (**i**, **n**, **q**, and **v**) reveal periods with large amplitude vibrations relative to background noise on the surface of the male. The male head was not available for analysis in the video capture by CAM 3 due to the angle of the elephant. By performing a short-time Fourier transform (6-bit window) of the male head signal, vibration spectrograms were generated from the different recordings (**g**, **l**, and **t**) showing a good match with audio spectrograms (**e**, **j**, and **r**)

**Fig. 11:**
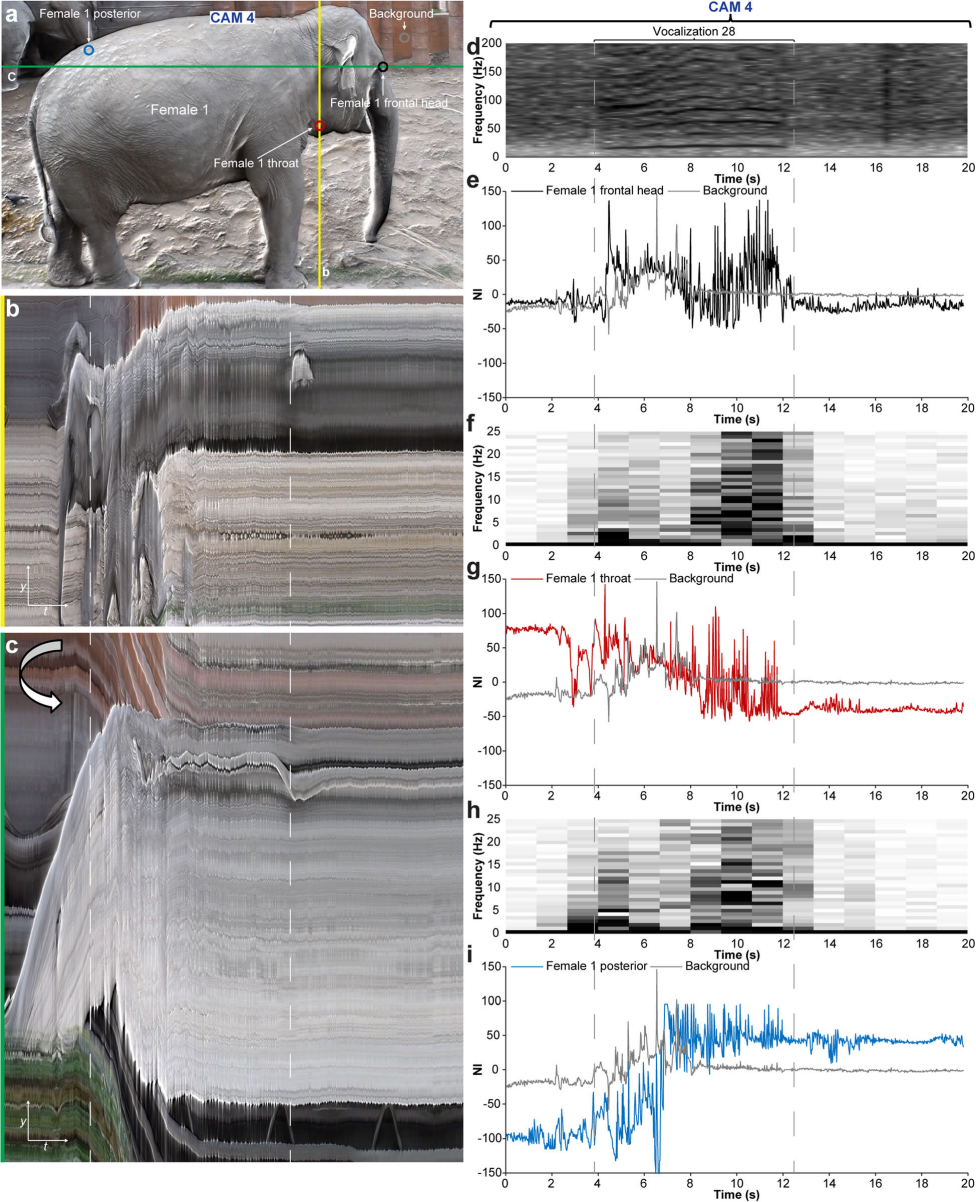
Single close-up video recording of one Asian elephant showing pronounced vibrations associated with vocalization. **a** Image from video recording by CAM 4. Image probes for vibration analysis are indicated on the image. **b**, **c** Vertical (**b**, yellow line in **a**) and flipped horizontal (**c**, green line in **a**) scan lines plots over time. The duration of one vocalization event (event 28 in Fig. 9) is highlighted by dashed lines. **d** Audio spectrogram. **e**–**i** Analysis of vibrations at different body positions during the video capture. Plots of normalized pixel intensity over time at the frontal head (**e**), throat (**g**), and posterior (**i**). **f**, **h** Vibration spectrograms from frontal head and throat signal. By performing a short-time Fourier transform (6-bit window) of the frontal head and throat signal, vibration spectrograms were generated (**f** and **h**) showing a good match with audio spectrogram (**d**)

Infrasound vocalization (< 20 Hz) in elephants represents a low-frequency, high amplitude signal that is difficult for humans to detect and localize by ear and in theory could be revealed with EVM processing of videographic material with a modest frame rate (> 40 FPS). To test if motion magnification could reveal subtle vibrations on different locations of the body of infrasound vocalizing elephants, we video-recorded three captive Asian elephants (*Elephas maximus* Linnaeus, 1758), one male (57 years of age) and two females (female 1: 18 years of age, female 2: 18 years of age) of which female 1 was 15 months pregnant at the time of recording. The elephants were video and audio recorded over a period of 931 s using five separate cameras (Fig. 9a; Additional file 13, first part) each recording with the same spatial resolution (1920 × 1080 px^2^) and temporal resolutions ranging from 25 FPS to 60 FPS (Table 1, Fig. 9b). During the main part of the recording (from 285 to 768 s), the three elephants were positioned in close proximity, facing the closed doorway to the inside enclosure where a caretaker was positioned and communicated with the elephants to encourage them to vocalize. The five cameras were positioned with maximal spacing given the constraints of the fenced enclosure (Fig. 9a–d). Three cameras (CAM 1–3) were stationary and two (CAM 4–5) were manually operated although stabilized on fence poles. During the last part of the recording (768–931 s), the elephant group split up and female 1 walked closely past the cameras. Audio spectrogram analysis revealed 28 distinct vocalization events with an infrasound component (Fig. 9e). Three vocalization events (Fig. 9e: vocalization events 3, 4, 28) were selected for EVM motion enhancements. Together, vocalization events 3 and 4 represent a sequence of vocalization (event 3), silence, vocalization (event 4), and silence (Fig. 10). The video recording made at the lowest temporal resolution (25 FPS, CAM 5) was inadequate for EVM enhancement of infrasound-related vibrations. However, EVM processing of recordings made at higher temporal resolutions (50–60 FPS, CAM 1–4) showed a match in time of high amplitude vibrations at the male head and male posterior to the audio spectrogram [compare Fig. 10e, j, o, r (audio spectrograms) with Fig. 10f, k, s (male head EVM processed signal), Fig. 10g, l, t (male head vibration spectrogram), and Fig. 10h, m, p, u (male posterior EVM processed signal); Additional file 13, second part]. On the other hand, there was no match in vibrations observed on the two females and the audio spectrogram [compare Fig. 10e, j, o, r (audio spectrograms) with Fig. 10i, n, q, v (female 1 and 2 posterior EVM processed signal)], suggesting that only the male was vocalizing with an infrasonic component during events 3 and 4. During vocalization event 28, female 1 was moving forward and only CAM 4 (manually operated) was in a position to record the entire vocalization event (Fig. 11). The EVM processed recording from CAM 4 showed a very precise match in time of high amplitude vibrations at the head, throat, and posterior to the audio spectrogram [compare Fig. 11d (audio spectrogram) with Fig. 11e, f (female 1 head EVM processed signal and vibration spectrogram), Fig. 11g, h (female 1 throat EVM processed signal and vibration spectrogram), and Fig. 11i (female 1 posterior EVM processed signal); Additional file 14]. In combination, the recordings of vocalization events 3, 4, and 28 demonstrate that EVM could be used to enhance and detect vibrations related to infrasound production at both anterior and posterior positions of the elephant body, and this could be used to determine which animal was vocalizing at a given time.

## Discussion

Eulerian video magnification and other video magnification techniques have been used in several recent studies with a medical scope to extract vital signs from videographic material based on widely available smartphone cameras [31–33], and even a remotely controlled camera mounted on a hovering aerial vehicle (drone) [34]. Additionally, video magnification has been used to enhance detection of fasciculations in people with amyotrophic lateral sclerosis based on videographic material [35] and enhance small motions in ultrasonographic material [36]. Although the medical potential of enhancing subtle color changes or motions in video material is obvious, the potential use of the method in experimental biology and comparative physiology should not be neglected. Thus, the aim of this study was to report how to apply Eulerian video magnification in a more basic experimental biology setting and to provide a series of examples of how signals of relevance, e.g., *f*_H_, respiration rate, PWV, and sound-induced vibrations, can be extracted from videographic material.

By examining the axolotl, we establish how several camera formats can be used for EVM processing in a laboratory setting (Fig. 3, Additional file 4) and demonstrate a two-step procedure to optimize band-pass filter and channel selection in the EVM procedure (Figs. 1a and 2a–j, Additional file 2). For reasons mentioned earlier, the axolotl is a well-adapted model for vital sign monitoring with EVM, and the method demonstrated its potential to extract *f*_H_ in both leucistic axolotls as well as in dark brown wild types (Fig. 4a–c) and in both single animal and multiple animal setups (Figs. 1, 2, 3, and 4, Additional files 1, 2, 3, 4, 5, 6 and 8). The simple physiology experiment in which *f*_H_ was measured with EVM under different physiological conditions (rest, exercise and anesthesia) demonstrates a laboratory setup in which *f*_H_ can be continuously monitored solely based on a camera signal, which in experimental cases of little or predicted variance in oxygen pulse, allows for a constant indirect evaluation of *MR* [18].

Under field conditions, video recording can be a useful method to document animal behavior. Heart and respiration rate may be welcome additional pieces of physiological information that, in some cases, can be extracted from videographic material. Whereas this possibility can come into consideration in prospective planning of future experimental setups, we also demonstrate that EVM can be used retrospectively on videographic material that was not produced with the intention of video magnification (Fig. 7). This can have relevance for comparative ecophysiology studies that require *f*_H_ measurements or *MR* estimations on non-disturbed animals in their natural environment. Given the availability of high-quality videographic material of wild animals that already exists in the form of nature and wild-life documentaries, informative studies may be made using EVM.

The selection of species that were used in the experimental series of this study, axolotl, zebrafish, human, sand lizard, giraffe, embryonic chicken, embryonic mouse, and elephant, reveal an important consideration for evaluating appropriateness of different animals for EVM, namely the lack of thick fur or plumage on the entire body surface or on select parts allowing for unobstructed view to the skin surface.

Measurements of *f*_H_ acquired with EVM in this study were validated, where possible, with other techniques (in axolotl and embryonic mouse with ultrasound, in human with palpation, and in embryonic chicken with direct observations of windowed eggs). Validation was not possible in the zebrafish, since any human interaction changes behavior and most likely *f*_H_ in this observant fish (personal observation), and electrocardiography (ECG) was inapplicable in an aquarium with multiple animals. However, the obtained *f*_H_ (EVM) ± 95% CI = 127.57 ± 7.87 BPM in the adult zebrafish corresponds well with, to our knowledge, the only other literature account of non-invasive measurement of conscious adult zebrafish resting *f*_H_ of 135–140 BPM in fish of the age of 50–100 days at 28 °C [37]. In this previous study, *f*_H_ was acquired by trans-illuminating individually isolated fish with a powerful light source, which could influence resting *f*_H_ via stress effects on autonomic *f*_H_ control. Thus, to the best of our knowledge, the present study provides the first account of post absorptive and undisturbed *f*_H_ in conscious, unrestrained and undisturbed adult zebrafish under normal light conditions and social setting of the animal. Although movements can be detrimental to EVM processing of videographic material, the zebrafish recordings demonstrate that even with rapid movements occurring at a high rate (Fig. 5f) it can be possible to extract *f*_H_ if this is out of the frequency range of the movements, and these displacements are small compared to the size of the animal.

Heart rate validation was also not possible in videographic material acquired from online sources that was not produced with the purpose of video magnification and *f*_H_ measurement (Fig. 7). The sand lizard (Fig. 7a–c) represents an ectotherm; thus, *MR* and *f*_H_ is highly dependent on ambient temperature and direct comparison with acquired *f*_H_ under unknown circumstances with literature derived *f*_H_ is problematic for this species. This point highlights an important consideration in designing EVM studies, namely the importance of initially ground truthing EVM processed video recordings of parameters of interest such as *f*_H_ and respiration rate with other methods such as ultrasound, direct observations or at least expected frequency ranges based on literature. The giraffe (Fig. 7d–g) on the other hand is endothermic, and under the assumption that the filmed animal is healthy and unstressed, the measured *f*_H_ of 42–45 BPM falls close to previously reported values of about 40 BPM in undisturbed quietly standing giraffes [38–40]. It has long been a topic of discussion how the giraffe regulates cerebral pressure and blood flow when changing posture from a head down situation (typically drinking) with the brain some 2 m below the heart to a head up situation with the brain 2–3 m above the heart in the matter of seconds and vice versa [39]. In a sophisticated experiment, Brøndum et al. [39] found that raising and lowering the head of anesthetized giraffes caused substantial changes in the blood flow and pressure in blood vessels of the neck but no significant change in *f*_H_ [mean *f*_H_ (head down) ± standard error of mean (SE) = 42 ± 4 BPM; mean *f*_H_ (head up) ± SE = 43 ± 4 BPM]. Although the measurements acquired with EVM of the video of a giraffe with the head down and up in this study support this observation (Fig. 7d–g), the data material for the head up posture (a single cardiac cycle) is too sparse to draw concrete conclusions.

In addition to *f*_H_ detection, we also demonstrate how EVM can be used to measure PWV in both a low blood pressure system such as the axolotl (Fig. 6a–d) and a high-pressure system such as the human being (Fig. 6e–n). To the best of our knowledge, no literature values for PWV in the axolotl dorsal aorta exist; however, the measured PWV in the human being [PWV (a. carotis communis) = 4.38 m/s; PWV(a. brachialis) = 3.62 m/s] are well within human reference values for PWV in the a. carotis communis and a. brachialis [41–43]. The acquisition of PWV from non-contact videographic material is interesting in a clinical setting since, for large elastic vessels, PWV both correlates non-linearly with arterial stiffness and can be applied to measure this important clinical parameter using the Moens–Korteweg equation, and PWV also correlates non-linearly with blood pressure, and thus, changes in PWV can be used to derive relative changes in blood pressure [44]. To obtain absolute blood pressure values, a subject-specific calibration is needed [43]. The applicability of EVM to acquire precise and accurate indirect measurements of blood pressure remains to be shown. However in this task, it is important to take account of the fact that the correlation of the two parameters is not valid in peripheral non-elastic vessels; thus, a previous attempt to measure pulse transit time with EVM at the neck and at the wrist of a bent arm [45], i.e., a span of vessels that contain peripheral non-elastic arteries, would not allow for PWV conversion to blood pressure.

The mid–late stages of embryonic heart development in birds and mammals represent an important timespan for the formation of structures that characterize high-performance four-chambered hearts. During this time, cushions form that later mature into valve leaflets and septation results in separate atria and heart chambers [30]. To understand what factors affect correct and incorrect heart development which can lead to congenital heart defects, it is important to be able to image the developing hearts of well-established avian and murine models at these stages. Micro-CT imaging provides a method to in vivo image mid–late-term developing hearts in chicken and in mouse, in direct three-dimensional acquisitions, in contrast to high-frequency ultrasound that currently only exists with linear array (2D) transducers, at a higher resolution than magnetic resonance imaging allows for, and with a larger depth of field than possible with optical coherence tomography. However, the minute beating hearts of the embryonic chicken and mouse are challenging to image since X-ray projections need to be allocated to specific cardiac phases to avoid image blurring. Electrocardiography can be applied to embryonic chicken [46]; however, electrode placement close to the embryo can result in image artifacts, and for the embryonic mouse, it is difficult to discriminate between the ECG from several embryos. We have previously used a non-enhanced optical signal acquired at the level of the beating heart or oscillating proximal vessels to prospectively gate micro-CT scans of chicken embryos at embryonic days 9 and 11 in windowed eggs (unpublished data). Here, we demonstrate that the less intense heartbeat signal at embryonic day 7 can be magnified to a level where it is detectable even through the eggshell, allowing for prospectively gated data acquisition with the micro-CT system (Fig. 8k–n, Additional file 11). Likewise, the heartbeat signal in embryonic mice in utero was detectable after EVM processing (Fig. 8o–v, Additional file 12), in theory allowing for a similar optical gating procedure of a micro-CT system if a protocol to administer a vascular micro-CT contrast agent to the embryonic mouse in utero is developed.

Vocalization in mammals span a frequency range of 9 Hz in blue whales [*Balaenoptera musculus* (Linnaeus, 1758)] [47] to 110 kHz in some bats [48]. Sound is a mechanical wave, and in those cases where sound production results in vibrations on the body surface of the source, these small motions can, in theory, be magnified in videographic data with EVM without aliasing, given that the data material has a temporal resolution of twice the frequency of the sound (Nyquist–Shannon sampling theorem), i.e., 18–220,000 FPS. In comparison, even the highest heart rates recorded in vertebrates, 1260 BPM (21 Hz) in blue-throated hummingbird [*Lampornis clemenciae* (Lesson, 1829)] [49] and 1511 BPM (25.18 Hz) in Etruscan shrew [*Suncus etruscus* (Savi, 1822)] [50], could in theory be video magnified with EVM without aliasing on videographic material with a modest frame rate just above 50 FPS. Thus, inexpensive camera equipment allows for *f*_H_ detection across all vertebrates that may be applicable for EVM, but without dedicated high-spatiotemporal resolution camera equipment, the video magnification of sound-induced vibrations is restricted to animals that vocalize at low frequencies with high amplitudes. All three extant species of elephants, the Asian elephant, the African bush elephant [*Loxodonta africana* (Blumenbach, 1797)], and the African forest elephant [*Loxodonta cyclotis* (Matschie, 1900)] vocalize and communicate with infrasound with fundamental frequencies typically in the range of 14–35 Hz with sound pressure levels as high as 103 dB (re 20 μPa) at 5 m from the source [51–54]. Infrasound production allows for long range communication between elephants, and it has been shown that the fundamental frequency of a rumble conveys information about the elephant that produced the sound, e.g., rumbles of females exhibit higher fundamental frequencies as compared to male rumbles [55], age is negatively correlated with fundamental frequency [56, 57], and individuals experiencing elevated levels of affect produce rumbles of a higher fundamental frequency [58, 59]. It has been demonstrated that myoelastic-aerodynamic flow-driven sound production can account for loud infrasonic vocalization in the African bush elephant without the need of active muscular contraction (purring) mechanisms [60]. Both mechanisms, however, would result in potentially detectable oscillations originating from the throat/head region propagating on the surface of the elephant body and possibly being attenuated at distal positions. In behavioral studies, it may be of interest to discern between individual vocalizing elephants at any given point in time. Although acoustic triangulation can be used in the field to localize a vocalizing elephant [61, 62], this method falls short when individual vocalizing elephants are in close proximity because of measurement error. Simple observation is frequently inadequate because calls often are not audible and vocalizations are not always linked to visible behaviors. In vocalization events 3–4 presented in this study, we were able to demonstrate, using multiple cameras, vibrational signals in the frequency range of infrasound with different intensities from three Asian elephants standing in close proximity in a suboptimal position for throat/head produced vibration detection (rear of animals facing the cameras, Fig. 9). Signal intensity was several folds higher in the male than the two females suggesting that the infrasonic component of the vocalization originated from the male in the group (Fig. 10, Additional file 13). Whether EVM is complementary to acoustic localization arrays for localization of a vocalizing elephant in a group in long range field conditions needs to be explored further using high-resolution cameras and high power telescopic lenses. In vocalization event 28 presented in this study, a single elephant was recorded vocalizing in close proximity to the camera (Fig. 11). A pronounced vibrational signal with a precise match in time with the acoustic signal could be detected with EVM both in the throat/head region and in the posterior region. No signal attenuation was observed between the proximal and distal locations (Fig. 11) suggesting that the vibrational signal travels well in the massive body of the elephant, meaning that the body position relative to the camera is of less importance when enhancing the subtle sound-induced surface vibrations in vocalizing elephants.

## Conclusions

In this methodology study, it is demonstrated how EVM can be used to acquire *f*_H_, respiration rate, pulse wave velocity, and infrasound vocalization-related vibrations from videographic material in a range of experimental setups. The method does not generate a signal where there is none, but enhances rhythmic signals, such as the heartbeat, relative to the noise level, so an inapparent signal can be localized, visualized, and recorded. The EVM methods work across a wide range of video qualities and spatiotemporal resolutions. Optimally, test objects are stationary, but movements out of the frequency range of the signal of interest can be acceptable. The importance of physiological measurements from undisturbed animals in their natural environment is becoming ever clearer in the fields of experimental biology and ecophysiology, which may in some cases limit the applicability of instrumentation of animals with recording devices. In this respect, EVM can, after a thorough validation with other techniques, provide a tool to prospectively or retrospectively extract signals of interest from a distance using simple inexpensive video recording equipment.

## Methods

### Animals and husbandry

Animals used for laboratory-based experiments in this study were the Mexican axolotl, the zebrafish, embryonic chicken, and mouse. Axolotl and zebrafish experiments were performed in Denmark and were approved by the Danish National Animal Experiments Inspectorate (Protocol# 2015−15−0201−00615 and Protocol# 2019-15-0201-00091, respectively). Axolotls were obtained from a commercial breeder (Exoterra GmbH, Germany) and were housed individually in plastic containers with a 10-cm water depth and a 930-cm^2^ surface area with regular water changes and a 12 h:12 h light to dark cycle. Animals were fed every second day with protein-enriched trout pellets. Axolotl anesthesia was obtained using 200 mg/L ethyl-4-aminobenzoate. Zebrafish were obtained from a commercial breeder (Lystrup Dyrecenter, Denmark) and were housed initially 61 animals together in a large 200-l aquarium with environmental enrichment via grating, brick, and stones; stocking density at the time of recording was 45 fish in the 200-l aquarium. Aquarium air stones provided aerated water, a thermostatic heating system set at 28 °C providing a measured temperature of 27.6 °C, and pump (EHEIM 200, Germany) with a 20-cm water depth and 4500-cm^2^ surface area with regular water changes and a 12 h:12 h light to dark cycle. Animals were fed daily with JBL NovoBel (tropical aquarium fish food, JBL, GmbH & Co.KG, Germany). Embryonic chicken experiments were performed in the USA (NY) where Institutional Animal Care and Use Committee (IACUC) approval does not apply for embryonic chickens. Eggs were obtained from local commercial breeder and were incubated with rocking at 37.5 °C in a water-saturated environment. All chicken embryos were terminated at embryonic day 9. The embryonic mouse experiment was performed in the USA (NY) where mice were bred at the lab animal facility under IACUC protocol #2008-0011. Mouse anesthesia was obtained by isoflurane inhalation (4% isoflurane in pure O_2_ for induction of anesthesia and 1.5% isoflurane in pure O_2_ for maintaining anesthesia), and the adult mouse was euthanized under anesthesia directly after video recording.

### Video material, camera, and audio

All video material from lab-based experiments was recorded using the built-in camera in a standard smartphone (Galaxy S6, Samsung, Seoul, South Korea) or a GoPro Hero 4 (GroPro Inc., San Mateo, CA, USA) compact camera applying spatiotemporal resolution settings as specified in Table 1. For the multi-camera recording of vocalizing elephants, the camera and built-in microphone of three different smartphones [Galaxy S6, Galaxy S6 Edge, and iPhone 6s (Apple Inc., Cupertino, CA, USA)] and two digital single-lens reflex cameras (Nikon D5300 with a AF-S DX NIKKOR 18–55 mm f/3.5–5.6G VR II lens and Canon Eos 300D with a Canon EFS 18-55mm f/3.5–5.6G Image stabilizer lens) were used.

Video material that was not produced with the intention of EVM processing was acquired from external online sources. The sand lizard video was produced by Ben Locke and was located on Flickr [63]; the giraffe video was produced by AliasAnimo and was located on YouTube [64]. Written permissions were obtained to apply the videos for EVM processing in the scope of the present study.

### Ultrasonography and micro-CT imaging

Ultrasonography on axolotl and embryonic mouse was performed with a Vevo 2100 system (FUJIFILM VisualSonics, Toronto, Ontario, Canada) with a 21-MHz transducer (MS250).

Ex vivo micro-CT imaging of a contrast agent (Microfil, FlowTech Inc., Carver, MA, USA) perfused axolotl was performed using a Scanco Medical XtremeCT system (Scanco Medical AG, Brüttisellen, Switzerland) with 1500 projections/180° and an isotropic voxel size of 41 μm, an X-ray tube voltage of 59.4 kVp, an X-ray tube current of 119 μA, and an integration time of 132 ms. In in vivo micro-CT imaging of embryonic chicken, an ex vivo imaging of embryonic mouse was performed using a eXplore CT 120 (GE Healthcare, Chicago, IL, USA) system with 1200 projections/180° and an isotropic voxel size of 25 μm, an X-ray tube voltage of 80 kVp, an X-ray tube current of 30 μA, and an integration time of 30 ms. For the in vivo embryonic chicken scans, an endoscopic camera (Qyuhe Mini USB Endoscope) was positioned in such a way that the egg surface could be viewed without the endoscope overlapping the trajectory of the X-ray beam of the micro-CT scanner. A flashlight placed directly at the end of the egg provided light for candling and heat during the micro-CT acquisition. Prospective gating was applied via custom-made peak detection software processing the real-time EVM-enhanced signal from the endoscope and signaling the micro-CT system a 5 V transistor–transistor logic false ECG pulse via a Bayonet Neill–Concelman connector.

### Eulerian video magnification procedure

The Matlab source code to perform EVM was released along with the original report of the procedure [10], and can be downloaded and used for research purposes from [65]. Additionally, an online freeware version exists for simple magnification procedures [66] as well as a commercial application package [66] and a software development package [66]. To demonstrate the relatively easy application of EVM in experimental biology, all videos in this study were processed using either the online freeware tool or the application package. To perform real-time EVM, the application package was needed. The EVM procedure of all videos in the study is outlined in the block diagram in Fig. 1a. Initially, a wide passband (frequencies of interest), a magnification type (color or motion magnification), and magnification level were selected. The resulting output video with a low SNR was used to extract an initial oscillation frequency, and this information was used to narrow the band-pass filter and reprocess the recording to produce a magnified video with a higher SNR. Frames were extracted from magnified videos using QuickTime Player version 7.7.9 (Apple Inc., Cupertino, CA, USA) and saved as three-dimensional stacks in TIF-format. Image analysis, i.e., channel splitting of RGB videos, selection of regions of interest, and signal extraction, was performed in ImageJ 1.5e (National Institutes of Health, USA). Unless otherwise specified, signal plots throughout figures are presented as normalized signal intensity (NI, i.e., signal intensity at any given time minus average signal intensity over the entire recording) over time or frequency. To decompose the signal into its constituent frequencies, Fourier transformation was used.

The most important step in the EVM procedure is the acquisition of an input video of adequate quality for subsequent processing. A list of prioritized considerations in the acquisition of videographic material for EVM or evaluation of non-EVM-dedicated material for the procedure is provided below:

1. *Stable camera.* A stable camera setup is all-important when recording videographic material for EVM processing. A moving camera results in a moving video frame, resulting in magnification of movements of all surfaces in the recording. Voice-operated cameras or delayed recording after touching the camera can be useful.
2. *Constant background.* The magnification technique does not discriminate on objects in focus and those in the background. Therefore, the background in the video should optimally be non-moving and preferably uniform in color.
3. *Reflections, shadows, and light source.* Reflection and shadows can interfere with EVM processing. If the experimental setup allows for it, reflections and shadows should be kept at a minimal level by adjusting the light source and structures in the vicinity of the scene. Some light sources oscillate with the utility frequency of the power grid, usually 50 or 60 Hz depending on location. For EVM processing in this frequency range, non-oscillating light sources should be used.
4. *Non-moving animal.* Eulerian video magnification is easiest to implement on non-moving animals. However, if movements appear at a frequency out of the frequency range of interest (e.g., the slow movement of an elephant producing infrasound at a higher frequency, Fig. 10 and Additional file 13), magnification can still be applicable.
5. *Choice of camera.* Eulerian video magnification can be used to videographic material produced on most currently available digital cameras. In this study, we primarily used smartphone cameras to demonstrate that the technique works with widely available camera type.
6. *Temporal resolution of the video (frame rate).* When trying to acquire an analog signal with a digital recorder, the frequencies that can correctly be obtained need to be below the Nyquist frequency, i.e., half of the sampling frequency to avoid aliasing (overlap of the signal). For example, a video recorded at 30 FPS one can only correctly magnify vibrations with a frequency < 15 Hz. Vibrations at higher frequencies may still be magnified so that they are visible on the magnified video, but frequency detection is unreliable in an under-sampled system.
7. *Spatial resolution of the video and color depth.* High-resolution, large color depth videos are desirable when trying to magnify subtle color changes or faint vibrations. Still, relatively low-resolution videographic material may still be useful [e.g., the low-resolution recording (640 × 480 px^2^) in Fig. 3u and Additional file 4 (last part) still allows for heartbeat magnification].
8. *Distance to object and optics.* Optimally, the object of interests should be close to the camera to detect small color changes and vibrations. Optics such as telescopic lenses can be used.
9. *View point.* It is important to consider the camera viewpoint of the object of interest. Some signals may be best observed from specific angles (e.g., a faint heartbeat signal is easier to detect from the ventral surface rather than the dorsal surface of most animals). In some cases, the experimental setup may dictate possible viewpoints that then need to be tested for the applicability with EVM.
10. *Duration of video.* Eulerian video magnification is a relatively processor demanding technique; therefore, the duration of videographic material should be limited to avoid unmanageable data acquisition.

### Statistics

Relevant types of *t* tests (paired/unpaired, one/two-tailed) were used to test for statistically significant differences between two groups. Relevant types of ANOVAs (repeated measure/mixed model, one-way/two-way) were used for omnibus testing of statistically significant difference between more than two groups. For post hoc tests of difference between groups, Tukey Honestly Significant Difference tests or Bonferroni corrected *t* tests were used. Significance level (*α*) was set at 0.05 unless specified in the case of the Bonferroni correction. For all statistical tests, *p* value was reported as actual number unless it was smaller than *α*/5 in which cases it was reported as *p* < α/5. Error bars in figures represent 95% confidence intervals.

## Supplementary information

**Additional file 5.** Heartbeat magnification in colored animal using Eulerian video motion magnification. Eulerian Video Magnified (motion) recording of one brown wild type axolotl axolotl. Dual trace video. Original video to the left and motion magnified video to the right. Note subtle movements of the front limbs in relation to the heartbeat. Video relates to Fig. 4a-c.

**Additional file 11.** Interactive two-phase model of embryonic chicken heart at day 7 of development. Three-dimensional interactive model of beating embryonic chicken heart at day 7 of development in two phases: Ventricular end-diastole and ventricular end-systole. The interactive PDF file should be viewed in Adobe Acrobat Reader 9 or higher. To activate the 3D feature click the model. Using the cursor it is now possible to rotate, zoom, pan the model, and in the model tree all segments of the model can be turned on/off or made transparent. The model tree is a hierarchy containing several sub layers that can be opened (+). Model relates to Fig. 8n.

**Additional file 13.** Eulerian video magnification of vibrations related to infrasound vocalization in Asian elephants. Overview of scene and magnified video recordings of three elephants. First part of the video provides an overview of the scene via an aerial photograph (used with permission from COWI) and by a three-dimensional reconstruction of the scene. In the second part four synchronous recordings of vocalizing elephants (vocalizing event 3 and 4) from CAM 1–4 that have been video magnified (motion) are displayed. Note that the recordings from each camera have a sound trace in which upper harmonics of low frequency vocalization are audible. Video relates to Figs. 9 and 10.

**Additional file 14.** Eulerian video magnification of vibrations related to infrasound vocalization in Asian elephant. Magnified video recording of one elephant close to camera. Video magnified recording of vocalizing elephant (vocalization event 28). Vocalization results in pronounced vibrations of the elephant (4–12 s in the video). Note that the video has a sound trace in which upper harmonics of low frequency vocalization are audible. Video relates to Fig. 11.

## Data Availability

The 232 source videos used for Eulerian video magnification in this study are available on Dryad Digital Repository in either MP4 or MOV format. To access source videos, the zipped file container is downloaded from https://datadryad.org/stash/dataset/doi:10.5061/dryad.s7h44j12q [67] and unzipped. The file name of each source video file is structured according to the following hierarchy: **1**_**2**_**3**_**4**_**5**_**6**_**7**_**8** where numbers refer to **1:** Species name; **2:** Animal#; **3:** Animal strain if relevant; **4:** Experiment specific information 1; **5:** Experiment specific information 2; **6:** Spatial resolution of video; **7:** Temporal resolution of video; **8:** Camera name, for example, ***(1)*** Amex_***(2)*** Axolotl1_***(3)*** Leucistic_***(4)*** Anaesthetized_***(5)*** LateralViewWithUltrasound_***(6)***1920x1080px^2_***(7)***30fps_***(8)*** SamsungGlaxyS6.
