# Supplementary figures and images for: Extracting physiological information in experimental biology via Eulerian video magnification
Source: BMC Biol. 2019 Dec 12;17:103. doi: 10.1186/s12915-019-0716-7 (PMC6907275; doi:10.1186/s12915-019-0716-7)

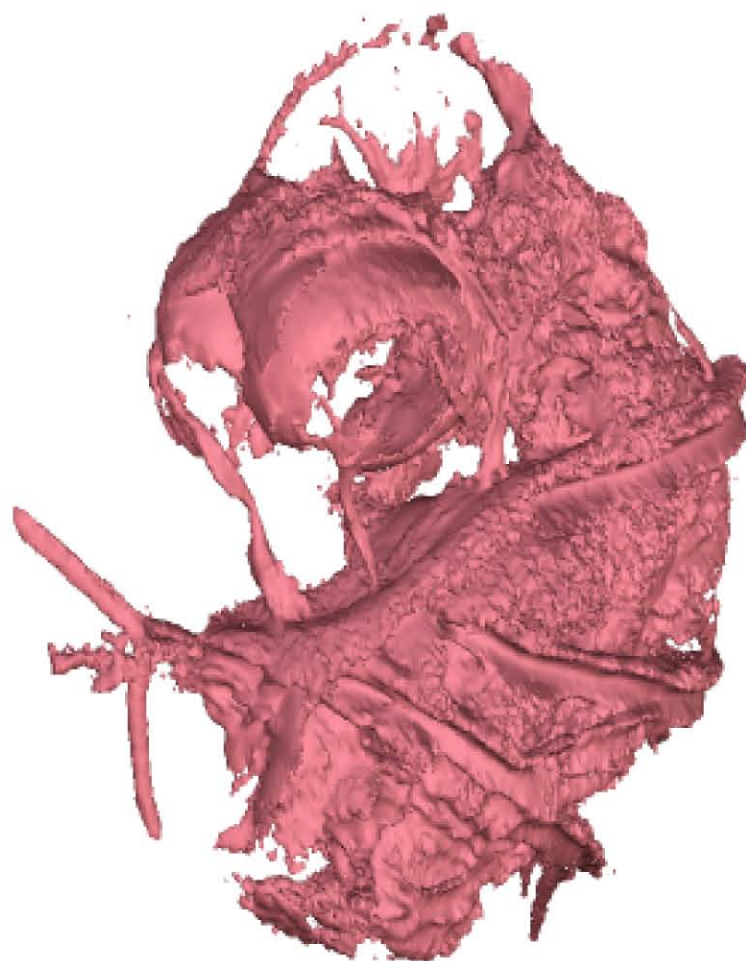

Supplement: Supplementary file 12 — Additional file 11. Interactive two-phase model of embryonic chicken heart at day 7 of development. Three-dimensional interactive model of beating embryonic chicken heart at day 7 of development in two phases: Ventricular end-diastole and ventricular end-systole. The interactive PDF file should be viewed in Adobe Acrobat Reader 9 or higher. To activate the 3D feature click the model. Using the cursor it is now possible to rotate, zoom, pan the model, and in the model tree all segments of the model can be turned on/off or made transparent. The model tree is a hierarchy containing several sub layers that can be opened (+). Model relates to Fig. 8n. [file 12915_2019_716_MOESM11_ESM.pdf]
